# High‐density intracranial recordings reveal a distinct site in anterior dorsal precentral cortex that tracks perceived speech

**DOI:** 10.1002/hbm.25144

**Published:** 2020-08-03

**Authors:** Julia Berezutskaya, Clarissa Baratin, Zachary V. Freudenburg, Nicolas F. Ramsey

**Affiliations:** ^1^ Brain Center, Department of Neurology and Neurosurgery University Medical Center Utrecht Utrecht The Netherlands; ^2^ Donders Institute for Brain, Cognition and Behaviour Radboud University Nijmegen The Netherlands; ^3^ Université Grenoble Alpes Grenoble Institut des Neurosciences Grenoble France

**Keywords:** ECoG, motor cortex, speech perception

## Abstract

Various brain regions are implicated in speech processing, and the specific function of some of them is better understood than others. In particular, involvement of the dorsal precentral cortex (dPCC) in speech perception remains debated, and attribution of the function of this region is more or less restricted to motor processing. In this study, we investigated high‐density intracranial responses to speech fragments of a feature film, aiming to determine whether dPCC is engaged in perception of continuous speech. Our findings show that dPCC exhibited preference to speech over other tested sounds. Moreover, the identified area was involved in tracking of speech auditory properties including speech spectral envelope, its rhythmic phrasal pattern and pitch contour. DPCC also showed the ability to filter out noise from the perceived speech. Comparing these results to data from motor experiments showed that the identified region had a distinct location in dPCC, anterior to the hand motor area and superior to the mouth articulator region. The present findings uncovered with high‐density intracranial recordings help elucidate the functional specialization of PCC and demonstrate the unique role of its anterior dorsal region in continuous speech perception.

## Graphical abstract

Berezutskaya et al. show that a distinct region within anterior dorsal precentral cortex tracks multiple auditory properties of perceived continuous speech. The region is distinct from the adjacent hand and mouth motor areas and has a unique role in speech processing.

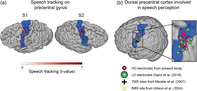

## 1 INTRODUCTION

It is widely known that speech perception engages a large network of brain regions. The involvement and functional role of some of these regions is better understood compared with other regions. In particular, various theories implicate importance of superior temporal, middle temporal and inferior frontal gyri in language processing (Friederici, 2012; Hagoort, 2013; Hickok & Poeppel, 2007). Activation of these regions has been extensively demonstrated for audiovisual and purely auditory speech perception (Crinion, Lambon‐Ralph, Warburton, Howard, & Wise, 2003; Wilson, Molnar‐Szakacs, & Iacoboni, 2008), for perceiving intelligible or noisy speech (Scott, Blank, Rosen, & Wise, 2000), and has been related to both semantic and syntactic processing (Rogalsky & Hickok, 2009).

When it comes to the sensorimotor involvement in speech processing, precentral gyrus and its neighboring sites, which we generally refer to as the precentral cortex (PCC), have also been shown to engage during both speech production and speech perception (Cheung, Hamilton, Johnson, & Chang, 2016; D'Ausilio et al., 2009; Pulvermüller et al., 2006; Skipper, Devlin, & Lametti, 2017; Skipper, van Wassenhove, Nusbaum, & Small, 2007). The area of PCC, whose language function is more comprehensively described, is the ventral portion of PCC. This region is otherwise known as the “face area” that controls facial and articulator movements necessary for speech production. Neuroimaging and electrophysiological studies have developed detailed maps of mouth articulators in the “face area” (Bleichner et al., 2015; Bouchard, Mesgarani, Johnson, & Chang, 2013; Chartier, Anumanchipalli, Johnson, & Chang, 2018). As the region also shows reliable activation during speech perception (Murakami, Restle, & Ziemann, 2011; Skipper, Nusbaum, & Small, 2005; Watkins, Strafella, & Paus, 2003), a number of theories have been proposed to explain how the primary function of the “face area” in speech production drives its responses to speech perception, including the motor theory of speech perception (Galantucci, Fowler, & Turvey, 2006, Liberman & Mattingly, 1985), the "analysis‐by‐synthesis" theory (Skipper, Nusbaum, & Small, 2006), the dual‐stream theory (Hickok & Poeppel, 2007) and others (see Skipper et al., 2017 for a review).

Recent studies on speech processing have also implicated a more dorsal region of PCC, adjacent to the cortex associated with upper limb motor control (Begliomini, Nelini, Caria, Grodd, & Castiello, 2008; Bleichner et al., 2015; Roland, Larsen, Lassen, & Skinhoj, 1980; Schellekens, Petridou, & Ramsey, 2018), which is often referred to as the “hand knob” (Yousry et al., 1997). Another adjacent region within the dorsal PCC region has been associated with the motor function of larynx (Dichter, Breshears, Leonard, & Chang, 2018; Simonyan & Horwitz, 2011) and with production of speech (Bouchard et al., 2013; Brown, Ngan, & Liotti, 2007; Dichter et al., 2018; Olthoff, Baudewig, Kruse, & Dechent, 2008), singing (Dichter et al., 2018), and vocalization in general (Brown et al., 2009).

In spite of its apparent specialization in motor control, dorsal precentral cortex (dPCC) and the neighboring cortex have been implicated in speech perception as well (Floel, Ellger, Breitenstein, & Knecht, 2003; Glanz et al., 2018; Keitel, Gross, & Kayser, 2018; Wilson, Saygin, Sereno, & Iacoboni, 2004). Some studies explain the activation of dPCC during speech perception through feedforward articulation‐to‐audio predictions (Meister, Wilson, Deblieck, Wu, & Iacoboni, 2007). Some other work points toward the facilitation function of dPCC in perception of speech under difficult conditions, where its activation compensates for the noisy input to the auditory cortex and aids in discrimination of speech sounds (Du, Buchsbaum, Grady, & Alain, 2014; Wilson & Iacoboni, 2006). Another line of research connects the involvement of dPCC in speech perception to cortical entrainment of rhythm, phrasal speech rates and encoding of the temporal structures in perceived stimuli (Bengtsson et al., 2009; Keitel et al., 2018). It is important to note that these studies focus on the neural activation to perceived speech in general, rather than responses to individual words or phrases semantically related to hand, face or body actions (see the theory of grounded cognition by Barsalou, 1999; Barsalou, Kyle Simmons, Barbey, & Wilson, 2003 and many related works, for example Hauk, Johnsrude, & Pulvermüller, 2004; Raposo, Moss, Stamatakis, & Tyler, 2009; Shtyrov, Butorina, Nikolaeva, & Stroganova, 2014).

Given that it is unclear how the dPCC region typically associated with motor planning and execution can be involved in speech perception, we here seek to elucidate such involvement. We report results of a rare opportunity to investigate the role of dPCC in speech perception from high‐density (HD) electrode grids placed in patients with epilepsy. These grids provide a unique combination of high temporal and spatial resolution that offers high detail of the underlying brain function (Jerbi et al., 2009). HD recordings obtained directly from the cortical surface preserve information often underrepresented or lost in other neuroimaging modalities (Berezutskaya, Freudenburg, Güçlü, van Gerven, & Ramsey, 2017; Dalal et al., 2009). Rather than employing specific tasks, the choice of which restricts evoked neural responses to constrained cognitive concepts (Brennan, 2016; Chen, Davis, Pulvermüller, & Hauk, 2013; Schmidt et al., 2008), we investigated data obtained while participants engaged in watching of a full‐length film. Such a naturalistic approach has been reported to be particularly beneficial in assessing cortical representation of a complex cognitive function such as speech processing (Glanz et al., 2018; Hamilton & Huth, 2018; Honey, Thompson, Lerner, & Hasson, 2012).

Data were collected from two patients implanted with HD intracranial grids (3 or 4 mm inter‐electrode distance) and two with standard (low‐density) clinical grids (10 mm inter‐electrode distance). The HD electrodes were placed over the sensorimotor cortex. The neural responses to speech and nonspeech film fragments were analyzed. We found that dPCC showed increased responses to speech compared with other auditory input. We were able to show that dPCC had the capacity to filter out background noise from the perceived speech signal and tracked various auditory properties of speech, such as its rhythmic phrasal structure, spectral envelope and pitch contour. None of these auditory properties was tracked as much in the nonspeech input. Importantly, we demonstrate that the location of the identified region is different from the hand motor and mouth articulator regions. The observed effects were prominent in HD data but were substantially weaker in the participants implanted with clinical intracranial grids. These results underline the specific function of dPCC in tracking of perceived speech and have direct implications on our understanding of the neural processes underlying continuous naturalistic speech perception.

## 2 MATERIALS AND METHODS

### 2.1 Participants

All participants were admitted for diagnostic procedures with medication‐resistant epilepsy. They underwent subdural electrode implantation with low‐density (LD) clinical grids to determine the source of seizures and test the possibility of surgical removal of the corresponding brain tissue. In two subjects, additional high‐density (HD) grids were placed over the sensorimotor cortex for research, after approving the procedure and signing the consent form. Research could be conducted between clinical procedures. All patients gave written informed consent to participate in accompanying electrocorticography (ECoG) recordings and gave permission to use their data for scientific research. The study was approved by the Medical Ethical Committee of the Utrecht University Medical Center in accordance with the Declaration of Helsinki (2013).

### 2.2 Film stimulus

A Dutch feature film “Minoes” (2001, BosBros Productions, www.bosbros.nl) was used as a stimulus for the film‐watching experiment. The film was 93 min long (78 min before credits) and told a story about a cat Minoes, who one day transforms into a woman. In her human form, she meets a journalist Tibbe. Together, they solve several mysteries involving their town and during their adventures eventually fall in love. The film was made in Dutch and was easy to follow for all ages. Patients reported that they had enjoyed watching the film.

### 2.3 ECoG experiment

Four patients (age 36, 30, 22, and 18, three females) watched the film. Two patients were implanted with grids in the left hemisphere, and two in the right. All patients had left hemisphere as language dominant, based on fMRI or the Wada test (Table 1).

**TABLE 1:** Electrode grid information for all participants (both HD and clinical low‐density)

Patient	No of electrodes	Grid hemisphere	Cortices covered	Handedness	Language dominance	Grid
S1	128	L	F, M, T	R	L (fMRI)	HD
S2	128	R	T, P, O	R	L (Wada)	HD, LD
S3	64	R	F, M, T, P	R	L (fMRI)	LD
S4	64	L	F, M, T, P	R	L (fMRI)	LD

*Note:* The table shows information about the number of electrodes, grid hemisphere, covered cortices, handedness, and language‐dominant hemisphere per patient.

Abbreviations: F, frontal cortex; fMRI, functional magnetic resonance imaging; L, Left; M, motor cortex; O, occipital cortex; P, parietal cortex; R, right; T, temporal cortex.

Two patients were implanted with HD grids over the sensorimotor region: S1 (128 contacts, 1.2 mm exposed diameter, inter‐electrode distance 4 mm, left sensorimotor cortex) and S2 (128 contacts, 1 mm exposed diameter, inter‐electrode distance 3 mm, right sensorimotor cortex). The suspected pathological regions in these patients did not extend to the sensorimotor region covered by the HD grids. This was clinically confirmed after implantation. Two remaining patients (S3 and S4) were only implanted with LD clinical electrode grids (2.3 mm exposed diameter, inter‐electrode distance 10 mm, between 48 and 128 contact points). LD grids had perisylvian coverage including frontal and motor cortices. Patient‐specific information about the grid hemisphere, number of electrodes, and cortices covered is summarized in Table 1.

In the experiment, each patient was asked to attend to the film displayed on a computer screen (21 in. in diagonal, at about 1 m distance). The stereo sound was delivered through speakers with the volume level adjusted for comfort for each patient. Due to the long duration of the film, patients were given an option to pause the film and quit the experiment at any time. In that case, the patient could continue watching the film at a later time starting from the frame they had paused on.

During the experiment, LD ECoG data were acquired with a 128 channel recording system (Micromed) at a sampling rate of 512 Hz filtered at 0.15–134.4 Hz. HD ECoG data were acquired with a separate system (Blackrock, Blackrock Microsystems) at a sampling rate of 2000 Hz filtered at 0.3–500 Hz. The film was shown using Presentation software (Neurobehavioral Systems), which allowed us to synchronize the film sound with the ECoG recordings. In addition, audio‐visual recordings of the room, patient, and computer screen were collected and used to confirm synchronization.

### 2.4 ECoG data processing

**FIGURE 1:**
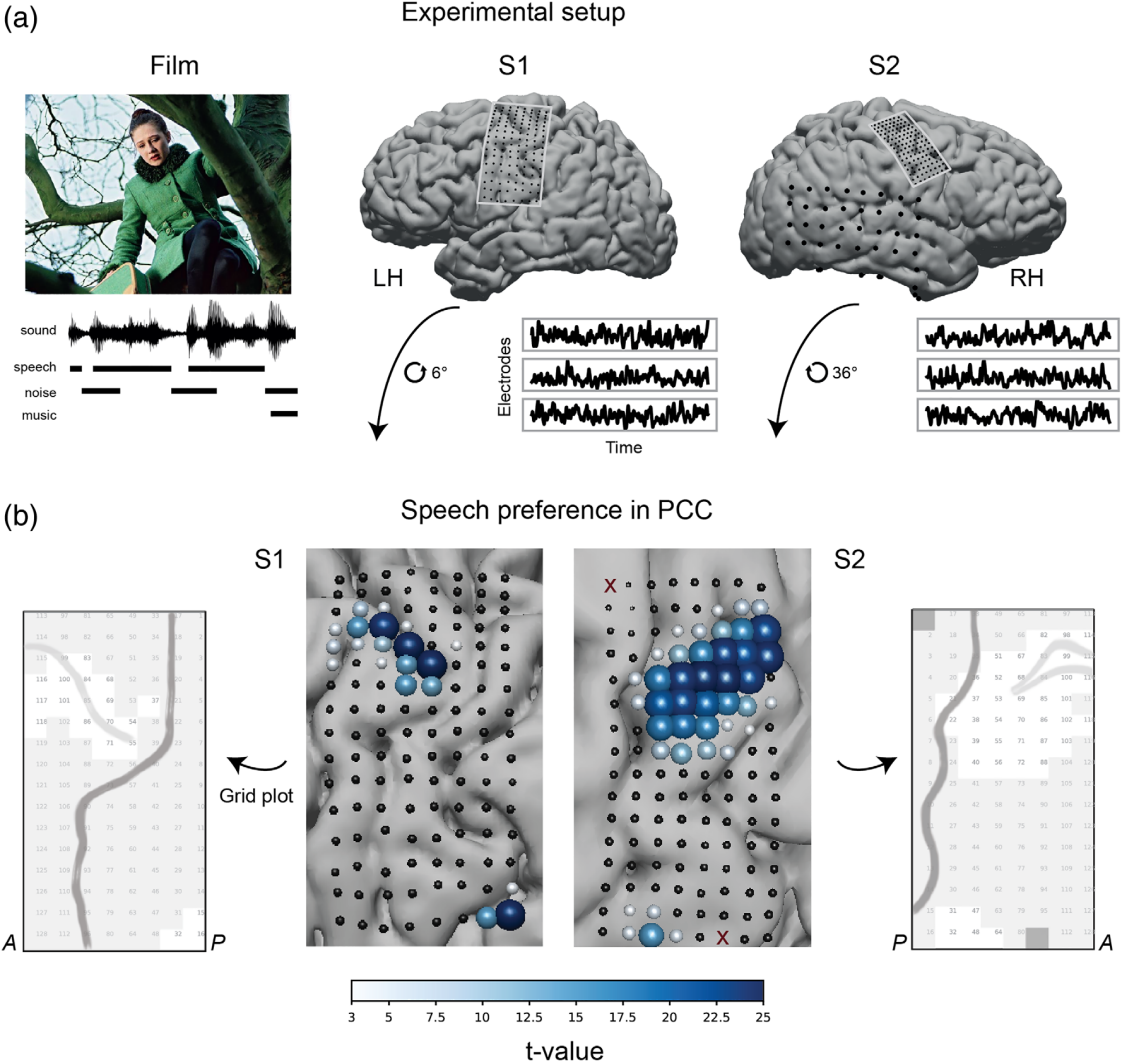
(a) Experimental setup. Two participants watched a full‐length Dutch feature film Minoes (2001). The soundtrack was annotated with fragments of speech, noise, and music sounds. During the experiment, each participant's neural responses were collected with HD ECoG grids placed over the sensorimotor cortex. A low‐density grid in S2 recorded from the temporal lobe. Recordings from a low‐density grid in S1 (not shown) were contaminated with epileptic seizures and were not analyzed. Example HFB time courses in three electrodes are shown per subject. (b) Results of the *t* tests comparing average HFB activity during speech and nonspeech (noise or music) fragments. HD grids were rotated for the visualization purposes. Each point on the grid is an ECoG electrode. Electrodes excluded from the analyses due to their flat or noisy signal are shown as dark red crosses (two electrodes in S2). Size and color of each electrode represent the t‐value. Positive t‐value represents higher average HFB activity during speech (*p* < .05, Bonferroni corrected for the number of electrodes). Next to the brain plots we show schematic grid plots, which are used for visualization in further analyses. Each square refers to one HD electrode. Numbers correspond to the electrode indices. Darker gray line shows the outline of the central sulcus. Lighter gray line shows the outline of the superior frontal sulcus. Grayed out electrodes in S2 show electrodes with either flat or noisy signal. These electrodes were excluded from all the analyses. A: anterior direction (towards the frontal lobe), P: posterior direction (toward the occipital lobe)

All electrodes with noisy or flat signal (based on visual inspection) were excluded from further analyses (two electrodes in S2, see Figure 1b). After applying a notch filter for line noise (50 and 100 Hz), common average referencing was applied per patient, separately for LD and HD grids. Data were transformed to the frequency domain using Gabor wavelet decomposition at 1–125 Hz in 1 Hz bins with decreasing window length (four wavelength full‐width at half maximum). Finally, high frequency band (HFB) amplitude was obtained by averaging amplitudes for the 65–125 Hz bins and the resulting time series per electrode were downsampled to 100 Hz. Electrode locations were coregistered to the anatomical MRI in native space using computer tomography scans (Branco et al., 2018; Hermes, Miller, Noordmans, Vansteensel, & Ramsey, 2010) and FreeSurfer (Fischl, 2012). The Desikan–Killiany atlas (Desikan et al., 2006) was used for anatomical labeling of electrodes in LD grids (closest cortical structure in a radius of 5 mm).

### 2.5 Linguistic annotation

The soundtrack of the film was extracted using Audacity software (Audacity Team). The stereo track was merged into a mono track and downsampled to 16 kHz. This audio track was used for linguistic annotation.

From the film production company we obtained film subtitles and the film script. These were used to produce a preliminary text‐to‐audio alignment in Praat (Boersma & Weenink, 2016). The alignment was created automatically by converting subtitle text into Praat annotations based on the subtitle time stamps. The subtitle text was compared against the script and corrected accordingly. Then, a number of undergraduate students were employed to correct the automatic text‐to‐audio alignment. Each student corrected the time markers of the subtitle text and created a tier with markers for onsets and offsets of individual words. Additionally, the students marked moments of overlap between speech and other sounds, such as music and audible noise. The students received detailed instructions regarding the waveform and spectrum properties of sound that could aid in determining the onsets and onsets of individual words. A trained linguist further verified their manual annotation. As a result, we obtained a linguistic annotation file with three tiers: subtitle text (a), individual word boundaries (b), overlap between speech and music or noise (c).

In addition, in the moments of the film with no speech present we annotated moments of presence of other sounds: music and various noises. These contained no overlap with speech and were used for extraction of nonspeech fragments.

### 2.6 Audio processing

The sound spectral envelope was extracted from the film soundtrack using NSL toolbox (Chi, Ru, & Shamma, 2005). We first extracted a sound spectrogram following the biological model of sound processing by the cochlea (Chi et al., 2005). The spectrogram was extracted at 8 ms frames along 128 logarithmically spaced frequency bins in the range of 180–7,200 Hz. We then averaged the spectrogram data over the frequency bins to obtain a 1D spectral sound envelope. The resulting spectral envelope was downsampled to 100 Hz to match the sampling rate of the ECoG HFB time courses.

In addition, for pitch‐related analyses we extracted pitch contour from the film soundtrack using an autocorrelation algorithm (Boersma, 1993) as implemented in Praat. We used the default parameters for pitch estimation.

In subsequent analyses we assessed the difference in neural processing of speech and nonspeech sounds. For this, we extracted a set of speech and nonspeech fragments of the sound track based on the manual linguistic annotation. We only included the annotations of clear speech (no overlap with music or noise) and speech that only slightly overlapped with music or noise, and the latter was rather stationary and soft (e.g., the last 100 ms of a fragment overlapping with beeping of a heart monitor or a sound of the rain, both being part of the film soundtrack). Each fragment was a continuous 4‐s long fragment of the soundtrack. In case of speech fragments, we allowed pauses between speech instances within a fragment of no longer than 500 ms. In total, this yielded 115 non‐overlapping 4‐s long speech fragments. Of note, these fragments did not cover all the clear speech material in the soundtrack but only a fraction (about 32%, or 7.67 min in total), in which every fragment was a 4‐s long continuous speech sequence with only pauses of no more than 500 ms. The fragments did not overlap. Then, we extracted a matching amount of nonspeech fragments. These contained no speech signal but included music, environmental sounds (thunder, street noises, birds chirping, animal cries, etc.), car, tool, and object sounds (placing dishes, typing, objects falling, phone ringing, etc.) and human‐made sounds (footsteps, clapping, gasping, laughing, etc.). Often sounds in nonspeech fragments overlapped, for example, music overlapped with footsteps, typing overlapped with thunder and so on. Many nonspeech fragments were heterogeneous and contained multiple different sounds within a 4‐s period.

Additionally, for further analyses on tracking of speech in noisy conditions we compared the amount of speech tracking in HFB responses in mixed sound track (what patients actually heard) and isolated speech track (speech‐only track obtained directly from the film company). The isolated speech track was processed the same way as the mixed sound track (extracted from the film as described above). Thus, we obtained the sound spectral envelope for the isolated speech track from the sound spectrogram and downsampled it to 100 Hz. In addition, for these analyses we selected a set of noisy speech fragments (i.e., with audible overlap of speech with music and noisy sounds, *n* = 63), based on the manual linguistic annotation. These fragments were also 4‐s long with pauses of no more than 500 ms. There was some overlap (17 fragments) between these 63 noisy fragments and the set of previously defined 115 speech fragments.

### 2.7 Preference to speech fragments in dPCC

Prior to the analyses on the HFB data we compared the overall sound intensity values between speech and nonspeech fragments to ensure that potential differences in HFB responses are not driven by the basic difference in sound intensity. The difference was assessed with an independent two‐sample *t* test on the raw sound intensity values (signal amplitude in time domain), averaged per individual fragment. Thus, we computed a t‐statistic on a vector of 115 speech sound intensities and 115 nonspeech sound intensities: *t* = 1.64, *p* = .1.

Having observed no significant difference in sound intensity between speech and nonspeech fragments, we compared average HFB amplitude values between the two types of fragments. For this, per electrode we averaged HFB responses over each 4‐s fragment and compared the vector of 115 HFB values in speech fragments against the vector of 115 HFB values in nonspeech fragments. The HFB data were z‐scored per electrode over the time points of all the used fragments (115 speech and 115 nonspeech fragments), however we also saw that performing *t* tests on the nonnormalized data led to the same statistical result. The *t* tests were conducted individually per electrode. The p‐values were computed parametrically and were corrected for multiple comparisons using Bonferroni correction for the number of electrodes per subject.

Because of the large number of electrodes per subject (*n* = 128 in both S1 and S2), the outcome of this analysis was used to limit the number of comparisons in further analyses. Thus, all further analyses involving HFB responses to the film were performed only in the subset of electrodes that showed preference to speech fragments (electrodes with significant *t* values from this analysis). This limited the number of multiple comparisons to 20 electrodes in S1 and 41 electrodes in S2.

All statistical testing for this and further analyses was conducted using *numpy* (Oliphant, 2006), *scipy* (Jones, Oliphant, & Peterson, 2001), *scikit‐learn* (Pedregosa et al., 2011) and *statsmodels* (Seabold & Perktold, 2010) libraries for Python.

### 2.8 Tracking of speech spectral envelope in dPCC

#### 2.8.1 Correlation to spectral envelope of speech

To assess the relationship between the sound spectral envelope and HFB data we computed the nonparametric Spearman correlation coefficient per electrode *e* (*ρ*_*e*_):

(1) $$\rho_{e}=\frac{\mathrm{cov}\left({\mathrm{rx}}_{e},ry_{e}\right)}{\sigma_{{\mathrm{rx}}_{e}}\sigma_{ry_{e}}}$$

where rx_e_ and ry_e_ are rank‐transformations of x_e_ (HFB response per electrode) and y_e_ (audio spectral envelope), respectively, and $\sigma_{rx_{e}}$ and $\sigma_{ry_{e}}$ are the *SD* of the rank variables. Both audio and neural data were characterized by highly skewed distributions with a long positive tail, and using rank‐transformation of the data allowed us to account for this skewness by capitalizing on the monotonic relationships in the data. As an alternative treatment, application of a log‐transform to both audio and neural responses followed by computation of Pearson correlations instead yielded essentially the same results.

The maximal correlation was determined per fragment from all biologically plausible lags in the range of −100 to 500 ms around the sound onset. The maximal correlation scores were Fisher‐transformed (*z*_*e*_) prior to further comparisons:

(2) $$z_{e}=\frac{1}{2}\ln\left(\frac{1+\rho_{e}}{1-\rho_{e}}\right)$$

Independent two‐sample *t* tests were used to assess the statistical difference between the average HFB correlation to the sound spectral envelope in speech and nonspeech fragments. The statistical significance was assessed parametrically and the *p*‐values were corrected for the number of electrodes in the analysis (20 electrodes in S1 and 41 electrodes in S2).

#### 2.8.2 Correlation to STG electrodes

First, per subject we identified a superior temporal cortex (STG) electrode (from the same HD grid in S1 and from a LD grid in S2) with the highest Spearman correlation to the audio spectral envelope. This correlation procedure was identical to the one described above. Then, the time course of the selected STG electrode was cross‐correlated to the dPCC electrodes (also through the similar correlation procedure, except that the maximal STG‐dPCC correlation was taken within the range of −200 to 200 ms). This range was chosen because the previously reported lags of brain response to speech perception was in the range of 200–400 ms for both PCC and STG (Cheung et al., 2016; Glanz et al., 2018; Kubanek, Brunner, Gunduz, Poeppel, & Schalk, 2013). Thus, the optimal lag between the two regions should be within the chosen range. Independent two‐sample *t* tests comparing correlations in speech and nonspeech fragments as well as the statistical significance were performed in the similar fashion as described above.

### 2.9 Filtering out of noise in speech fragments

Only the previously extracted noisy fragments (*n* = 63) were used for this analysis (see Audio processing section). Spearman cross‐correlation and paired sample *t* tests were used to compare HFB correlations to the speech spectral envelope in isolated and mixed sound tracks. The procedure followed the previously described Spearman correlation and *t* test pipeline. However, instead of comparing correlations during speech and nonspeech fragments, we compared correlations to the speech spectral envelope in isolated speech and mixed sound tracks and therefore used paired sample *t* tests. Apart from that, all procedures were identical to the correlation and *t* test procedures described above. The range of −100 to 500 ms was used to identify the maximal correlation to the sound envelope.

### 2.10 Capturing of the rhythmic phrasal structure of speech in dPCC

#### 2.10.1 Following of the phrasal grouping patterns in a continuous stream of speech

**FIGURE 2:**
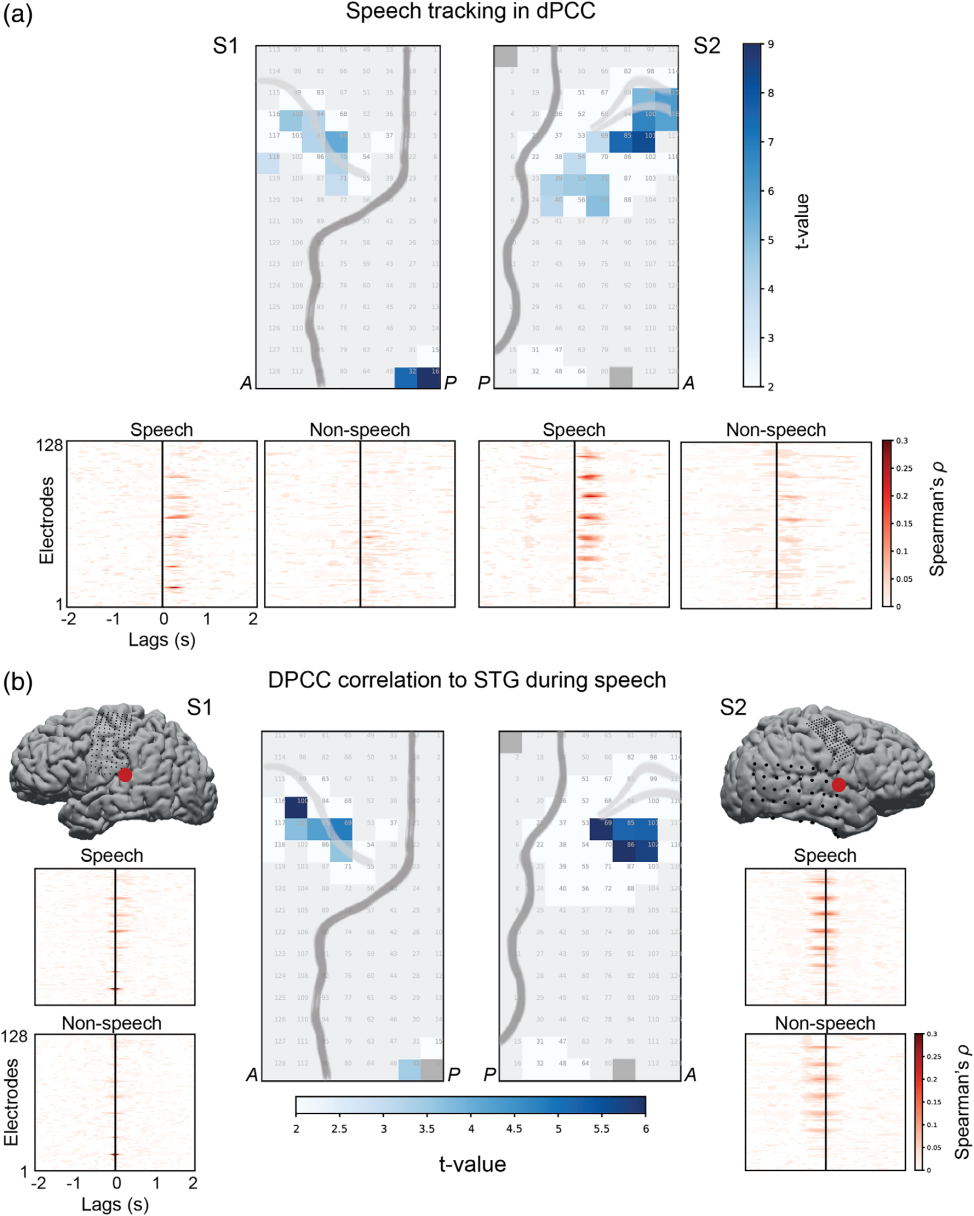
Speech tracking in dPCC. (a) Top panel shows results of the *t* tests comparing the amount of correlation to the spectral envelope of the audio during speech and nonspeech fragments (*p* < .01, Bonferroni corrected for the number of electrodes). Bottom panel shows individual cross‐correlation profiles per electrode separately for speech and nonspeech fragments. The x‐axis represents cross‐correlation lags (in seconds), where positive lags indicate that audio precedes the neural activity. The cross‐correlation profiles were averaged over all fragments per condition: 115 speech and 115 nonspeech fragments. For statistical testing, per electrode the maximal cross‐correlation value was selected in the range of −100 to 500 ms per individual fragment of each condition. The correlation values were Fisher‐transformed and fed into an independent two‐sample *t* test per electrode. The results were Bonferroni corrected for the number of *t* tests (=number of electrodes). (b) Center plots show results of the *t* tests comparing the amount of correlation to a STG electrode (that best correlated to the spectral envelope of the audio) in speech and nonspeech fragments (*p* < .01, Bonferroni corrected for number of electrodes). Side plots show individual cross‐correlation profiles per electrode. The procedure for conducting *t* tests on the correlation values was identical to the previous analysis with correlations to the spectral envelope of the audio

To determine whether HFB responses in dPCC followed phrasal grouping patterns in speech we first constructed a binary speech ON/OFF vector. All previously used speech fragments (*n* = 115) were concatenated. Using the manual linguistic annotation we assigned a value of 1 to all time points during speech and a value of 0 to all time points where speech was absent (= pauses in a continuous stream of speech). Given that we previously observed a time lag in HFB tracking of the audio, prior to the linear fit we shifted the HFB response by a lag of the maximal HFB‐spectral envelope correlation (a positive shift of up to 500 ms, see cross‐correlation plots in Figure 2a). The shift was applied individually per speech fragment and per electrode. Then, for the linear fit, both HFB responses and the binary speech ON/OFF vectors were further concatenated across all speech fragments. Thus, we fitted a single regression model for all data rather than fitting an individual regression model per speech fragment.

A linear regression was used to predict z‐scored HFB responses per electrode e (y_e_) using the binary speech ON/OFF vector (x):

(3) $$y_{e}={\beta_{e}}^{Τ}x+\varepsilon_{e}$$

where $\varepsilon_{e}\sim N\left(0,\sigma \right)$.

The ordinary least squares solution was used. The statistical significance of the fit was assessed using F‐tests (with the null hypothesis that all regression *β*‐weights were equal to zero) and permutation testing for determining the chance threshold of the F‐statistic. During the permutation testing we permuted the order of the speech fragments prior to their concatenation 10,000 times and each time fitted a new linear regression on the permuted speech ON/OFF vector. Then, we compared the F‐statistic of the actual fit to the 99.999th percentile of the permutation distribution, which corresponds to a chance level of .001. The significance testing procedure was repeated per electrode.

As a control analysis, the analogous linear fit was computed on concatenated HFB data from nonspeech fragments and a sound ON/OFF binary vector. The sound ON/OFF binary vector was obtained using Praat function Intensity to Silences, that automatically labels the moments of sound and no sound in the audio using an intensity threshold *I*_max_ − 35 dB, where *I*_max_ is the maximal intensity in dB. This intensity threshold was the default value. We found that in our audio this threshold produced best estimation of silence versus sound periods.

Similar to the fit on speech fragments, we first shifted the HFB responses during nonspeech fragments to the lag of the best HFB‐spectral envelope correlation. The plots in Figure 2a indicated significantly lower correlation during nonspeech fragments, but it still appeared to be above zero, especially in S2 and overall was restricted to the lag of up to 500 ms. Then, the data were concatenated over nonspeech fragments and the linear fit was computed in the same way as described above.

The difference in the *β*‐weights over the dPCC electrodes between the fit for the speech fragments and the fit for the nonspeech fragments was assessed using an independent two‐sample *t* test per subject.

### 2.11 Capturing of speech pitch in dPCC

Given that both pitch contour and the spectral envelope reflect spectrotemporal properties of speech, prior to the analyses on the neural data we assessed the amount of shared information between the two auditory features. For this, we computed correlations between pitch contour and the spectral envelope in speech and nonspeech fragments separately. We calculated both Pearson and Spearman correlation coefficients, and the results were comparable between the two. For consistency with the previous analyses we reported the Spearman correlation values in Figure 5a. The amount of correlation was significant in both speech and nonspeech conditions (as tested with one‐sample *t* tests on the Fisher‐transformed correlations). The difference in correlation between speech and nonspeech fragments was assessed using an independent two‐sample *t* test on the Fisher‐transformed data. In addition, we also computed the amount of correlation between the spectral envelope and pitch contour for the noisy speech fragments, using isolated speech‐only sound track and the mixed sound track. The difference in correlation between the two tracks was also assessed with a paired two‐sample *t* test on the Fisher‐transformed correlation data.

For the analyses on the neural data, we aimed to account for the interactions between the spectral envelope, rhythmic phrasal structure and pitch contour. For this, we used residuals of the previous analysis fitting the binary ON/OFF speech vector (rhythmic phrasal structure) to the HFB responses and computed partial correlations of the HFB residuals with pitch contour and the spectral envelope. Per ECoG electrode (same selection of electrodes as in all previous analyses), we computed the partial Spearman correlation with pitch while accounting for the spectral envelope data (a) and with the spectral envelope data while accounting for pitch contour (b). The analysis was only performed on data from the speech fragments.

### 2.12 Analysis of residual HFB responses to speech and nonspeech fragments

Finally, we assessed the difference in the HFB responses during speech and nonspeech fragments by taking into account the gained knowledge about HFB tracking of the auditory properties of the input audio signal. We performed an ordinary least squares fit to predict the HFB responses based on all previously used auditory properties (spectral envelope, rhythmic phrasal structure, and pitch contour). The fit was computed separately for speech and nonspeech fragments. In nonspeech fragments, the “rhythmic” binary vectors also captured pauses and similar to the speech condition represented the sound being ON or OFF. Pitch contour and spectral envelope were calculated the same way as for the speech fragments.

HFB residuals of the fit using auditory properties were compared between speech and nonspeech conditions. For completeness, we also included comparisons with the original HFB responses to speech and nonspeech fragments (HFB data prior to the fit, same data as used in the first *t* test analysis comparing average responses to speech and nonspeech fragments). HFB data were z‐scored prior to the fit. Because we aimed to compare the average HFB amplitude between the original data (“full”) and the same data after regressing the auditory properties (“residuals”) in both conditions (speech and nonspeech) we refrained from using parametric approaches such as a one‐way analysis of variance (ANOVA) test. It seemed logical to assume that in the case of a successful fit, the original data and the residuals would not have equal population variances. Instead, we opted for a nonparametric test based on ranked transformations of the data, such as a Kruskal‐Wallis test. In case of the rejection of the null hypothesis that all groups had equal means, we performed post hoc tests determining which groups of data showed significant difference in means while accounting for multiple comparisons (nonparametric post hoc Dunn's tests). In addition, since the groups were clearly organized along two factors (type of fragments: speech and nonspeech, and type of used data: full or residual HFB responses), we aimed to investigate the main effects of each factor and their interaction. To account for the violation of the assumption about equal population variances and to follow the logic of the Kruskal‐Wallis and Dunn's tests, we performed a two‐way factorial ANOVA analysis (which was simply equivalent to a linear regression using categorical factor variables) on the rank‐transformed neural data.

### 2.13 Functional specialization in PCC

#### 2.13.1 Testing interference from visual hand perception

All speech (*n* = 115) and nonspeech (*n* = 115) fragments used in the analyses were annotated with respect to hand presence and movement in the movie frames. For annotation we used ELAN software (Brugman, Russel, & Nijmegen, 2004), which unlike Praat supports a video stream. We went through every frame corresponding to speech and nonspeech fragments and annotated it with hand movement using a three‐level scale: 0—no hand presence, 1—hands are visible but there is no movement, 2—clear hand movement. A *χ*^2^ analysis was employed to test interaction between speech and hand variables across the fragments. The main *χ*^2^ test reported in the Results assessed interaction between two levels of the speech variable (“speech present” and “speech absent”) and three levels of the hand variable (“hand moving,” “hand present,” and “hand absent”). In addition, we performed another *χ*^2^ analysis with a simplified hand variable that contained only two levels (“hand present” and “hand absent”) by replacing all “hand moving” annotations with “hand present” annotations. There was no interaction between hand and speech variables as a result of this analysis either: *χ*^2^(2, 609) = 0.97, *p* = .32.

In addition, we also used the hand movement annotation (the three‐level scale one) as a covariate in two previous analyses: the speech preference analysis (*t* test on average HFB amplitude in speech vs. nonspeech fragments) and the tracking of the spectral envelope analysis (cross‐correlation of HFB to the spectral envelope). For this, we constructed a vector of hand movement/presence/absence values per fragment (both speech and music fragments were used, 230 fragments in total). The data were concatenated across all fragments and the least ordinary squares fit was applied to predict HFB data using the hand regressor values. The obtained residuals of the linear fit were used to repeat the speech preference and the tracking of the spectral envelope analyses. For both analyses new t‐statistics (comparing speech and nonspeech conditions) were obtained using residual HFB data. These updated t‐statistics were compared against the original t‐statistics (obtained from HFB data without regressing out the hand annotation). The statistical comparisons of the original and updated t‐statistics were performed using nonparametric two‐sided Wilcoxon signed‐rank tests. Only electrodes with significant original t‐statistics were used in these comparisons.

#### 2.13.2 Relation to the hand motor and mouth articulator localizers

Both HD patients performed separate localizer tasks to identify cortex involved in hand motor and mouth motor execution. The hand motor task has a finger movement task with a randomized event‐related design. The task was previously used with fMRI and ECoG to obtain cortical representation of finger movement (Siero et al., 2014). Each patient was instructed to flex the thumb, index or little finger of their right hand depending on the cue. Each trial consisted of two flexions of one finger. During “rest” trials patients were instructed to remain still. The data from both subjects were preprocessed (bad channel rejection, line noise removal) and responses in HFB (65–125 Hz) were extracted. The data from three finger movement conditions (“thumb,” “index,” and “little finder”) were all treated as the single “move” condition. The “move” condition trials were compared against the “rest” trials using a signed *r*^2^ statistic (Figures 7a and S2). The reported *r*^2^ values were significant at *p* ≪ .001 in each subject.

The mouth articulator task also had a randomized event‐related design. The task was previously used with fMRI and ECoG participants to identify cortical sites involved in articulation (Bleichner et al., 2015; Salari et al., 2019). Each patient was instructed to move different parts of their mouth involved in articulation: lips, tongue, jaw, or larynx depending on a cue. On the cue “lip”, the patient performed a lip protrusion movement; on the cue “tongue”, the patient moved their tongue from left to right behind their teeth; on the cue “teeth clench”, the patient clenched their teeth; and on the cue “*mmmh*”, they produced the corresponding sound activating their larynx. During “rest” trials patients were instructed to remain still. The data from both subjects were preprocessed (bad channel rejection, line noise removal) and responses in HFB (65–125 Hz) were extracted. The data from four articulator movement conditions (lips, tongue, jaw, and larynx) were all treated as the single “move” condition. The “move” condition trials were compared against the “rest” trials using a signed *r*^2^ measure (Figures 7a and S3). The reported *r*^2^ values were significant at *p* ≪ .001 in each subject.

### 2.14 Reproducibility of results with LD grids

In addition to the main analyses on HD recordings, data from two patients who were only implanted with LD clinical grids were analyzed. Similar to the analyses on HD data, first, HFB responses were extracted for speech and nonspeech fragments. The data were averaged per fragment and compared across speech and nonspeech fragments using *t* tests. Next, we calculated Spearman cross‐correlations with the sound spectral envelope and cross‐correlations with STG electrodes. The procedures were identical to the ones carried out on HD data.

## 3 RESULTS

In this study, we investigated the involvement of dPCC in naturalistic speech perception using HD intracranial electrode recordings. Two participants (S1 and S2) implanted with HD grids over PCC watched a full‐length feature film (Figure 1a). We then analyzed their brain responses in 65–125 Hz (high frequency band, HFB) (Crone, Miglioretti, Gordon, & Lesser, 1998; Ray, Crone, Niebur, Franaszczuk, & Hsiao, 2008) in relation to the speech fragments of the film. First, in each subject we identified a set of electrodes in dPCC with significantly higher HFB responses to speech compared with nonspeech fragments (music, noises, animal cries, etc.). Then, we investigated the relationship between the responses of these electrodes and various auditory properties of speech, such as the speech spectral envelope (associated with loudness, pitch, timbre, and rhythm), its rhythmic phrasal pattern, and pitch contour. We found significant amount of neural tracking of these auditory properties. We also examined neural tracking of noisy speech fragments and found that dPCC electrodes had the ability to filter out background noise during perception of speech. Interestingly, the effects reported here were strong in participants with HD electrode grids, but were substantially less clear in participants with LD electrode grids.

### 3.1 Preference to speech fragments in dPCC

First, we aimed to determine whether any parts of PCC showed larger response amplitude during speech perception compared with perception of other sounds. Because PCC is not generally considered as part of the sound processing network, we did not additionally evaluate whether it generally exhibited a higher response to sound compared with silence. Since our goal was to determine speech‐specific response in PCC, we considered nonspeech sounds as a baseline for our comparison. Thus, for our speech/nonspeech comparison, we extracted 115 four‐second‐long fragments of each group (speech and nonspeech fragments) and compared the average HFB responses associated with each group. The nonspeech fragments contained various sounds, such as music, environmental noises (e.g., thunder, animal cries etc.), technical noises (e.g., car noises, phone ringing etc.), footsteps, clapping, etc. HFB responses were averaged per fragment, and the groups were compared using independent samples *t* tests (see Methods for details).

Prior to the analysis we determined that the selected speech and nonspeech fragments did not differ in terms of their overall sound intensity (sound amplitude in time domain): *t* = 1.64, *p* = .1 (Figure S1). Then, the *t* tests on the average HFB responses in speech and nonspeech fragments were conducted per electrode. They showed that 20 electrodes in S1 and 41 electrodes in S2 on average exhibited higher responses to speech: *t*_*S*1_ ranged from 3.9 to 27.85 and *t*_*S*2_ ranged from 4.91 to 27.74 (*df* = 228) at *p* < .05, Bonferroni corrected for the total number of electrodes (Bonf. cor., Figure 1b). The reported ranges include only significant electrodes, which were found anterior to the central sulcus in both subjects and corresponded to dPCC. The electrode locations formed consistent clusters in both subjects. Two more electrodes with a significant effect in S1 were located in STG and four more electrodes with a significant effect in S2 were located in ventral PCC. We used the outcome of this analysis (significant *t* values) to restrict the number of electrodes used in further analyses to 20 electrodes in S1 and 41 electrodes in S2.

Thus, we have observed that a subset of electrodes in dPCC showed preference to speech over other auditory input. To understand the nature of the dPCC response to speech better, we investigated its activity in relation to various perceptually relevant properties of speech. First, we examined whether dPCC tracked the overall shape, or envelope, of the spectrotemporal speech signal that is relevant for perception of consonants and vowels and overall speech intelligibility. Then, we investigated dPCC responses to speech in noisy conditions, which is relevant for perception of speech in mixed auditory input. Third, we examined the neural activity related to the rhythmic structure of speech that is relevant for parsing of continuous speech input into meaningful groups. Finally, we investigated the encoding of pitch contour, which is relevant for perception of intonation changes and speaker identification.

### 3.2 Tracking of speech spectral envelope in dPCC

#### 3.2.1 Correlation to spectral envelope of speech

To begin with, we focused on a slow‐varying spectrotemporal feature of the speech sound, the spectral envelope. The spectral envelope is computed from the speech signal transformed to the time‐frequency domain. It is computed for each time point as the signal energy averaged over all frequencies relevant to speech (180–7,200 Hz, Chi et al., 2005). It captures perceptually relevant characteristics of consonants and vowels and the temporal structure of speech (Ter Keurs, Festen, & Plomp, 1992), preserves spectral information reflecting speaker identity (Carey, Parris, Lloyd‐Thomas, & Bennett, 1996; Kitamura & Akagi, 1995) and is important for overall speech intelligibility (Arai, Pavel, Hermansky, & Avendano, 1996; Ter Keurs, Festen, & Plomp, 1993). Here, we tested whether dPCC electrodes responded to the speech spectral envelope by cross‐correlating it to HFB responses in the electrodes that displayed significant effects in the previous *t* test comparing responses to speech and nonspeech fragments. Spearman cross‐correlation (*ρ*) was performed per speech fragment (*n* = 115) and compared with the control condition (nonspeech fragments) to test whether tracking of spectral envelope was stronger in speech than nonspeech fragments.

A subset of previously defined electrodes (from the *t* test above: 20 in S1 and 41 in S2) showed higher correlation to the spectral envelope in speech (${\bar{\rho }}_{S1}=.25\pm .04$ and ${\bar{\rho }}_{S2}=.27\pm .03$) compared with nonspeech fragments (${\bar{\rho }}_{S1}=.16\pm .01$ and ${\bar{\rho }}_{S2}=.17\pm .03$), as indicated by *t* tests (on the Fisher‐transformed *ρ*‐values) at *p* < .01, Bonf. cor. (Figure 2a). The reported values show mean *ρ*‐values and *SD* over all significant electrodes (*ρ*‐values were first averaged over all speech or nonspeech fragments per electrode). The effect was significant for 7 of 20 electrodes in S1 and 13 out of 41 electrodes in S2. All electrodes that showed significant tracking of the speech spectral envelope (compared with nonspeech baseline) were localized in the dorsal portion of precentral gyrus, except for two STG electrodes in S1.

In addition, we observed that the highest *ρ*‐values typically fell in the range of 200–400 ms after sound onset (Figure 2a, bottom panel) suggesting that there was a positive ≈300 ms lag of speech tracking in dPCC.

#### 3.2.2 Correlation to STG electrodes

To further investigate the neural tracking of the speech spectral envelope in dPCC, we assessed the relationship between dPCC and electrodes directly involved in auditory processing, such as STG electrodes. An increased correlation between dPCC and STG during speech perception could be due either to elevated communication between the two regions during speech perception or their independent involvement in processing of speech fragments. These can be distinguished by examining the lag of correlation as that can reveal a latency between the regions. A lag would indicate dependency, while no lag would indicate that both regions process speech input in parallel to each other. Spearman cross‐correlation scores between dPCC and STG electrodes were calculated during speech fragments and compared with the cross‐correlation scores in nonspeech fragments with *t* tests. For this, in each subject we first identified a single STG electrode with strongest speech tracking (i.e., highest correlation to the speech spectral envelope: ${\bar{\rho }}_{S1}=.34\pm .14;\;{\bar{\rho }}_{S2}=.32\pm .16$) and then cross‐correlated its time course with all previously selected dPCC electrodes.

*T* tests between Fisher‐transformed dPCC‐STG correlations in speech and nonspeech fragments resulted in a set of dPCC electrodes with higher correlation to STG during speech (${\bar{\rho }}_{S1}=.29\pm .02$ and ${\bar{\rho }}_{S2}=.35\pm .02$) compared with the nonspeech condition (${\bar{\rho }}_{S1}=.22\pm .02$ and ${\bar{\rho }}_{S2}=.25\pm .03$, Figure 2b). The reported values show mean *ρ*‐values and *SD* over all significant electrodes (*ρ*‐values were first averaged over all speech or nonspeech fragments per electrode). The location of these electrodes was similar to the previous analysis (cross‐correlation to speech envelope) and restricted to the dorsal precentral gyrus (except for STG electrode 32 in S1). The electrode set included 5 out of 20 electrodes in S1 and 5 out of 41 electrodes in S2. The lag of maximal dPCC‐STG correlation varied across the fragments, fluctuating mostly around zero, which indicated that both regions likely tracked speech input parallel to each other.

### 3.3 Filtering out of noise in speech fragments

Given the observed presence of speech tracking in dPCC, we addressed the question of how tracking of a continuous stream of speech is affected by additional sounds that often occur in natural situations. The question lends itself to being addressed since multiple scenes in the film contained dialogs with overlapping music, multiple people talking at the same time, sound effects, and distracting background noise (“noisy” fragments). We investigated how the responses in dPCC were affected by the mix of sounds in the speech stream. In particular, we assessed whether activity in dPCC evidenced responding to speech specifically or to the composite auditory input (speech plus other sources).

**FIGURE 3:**
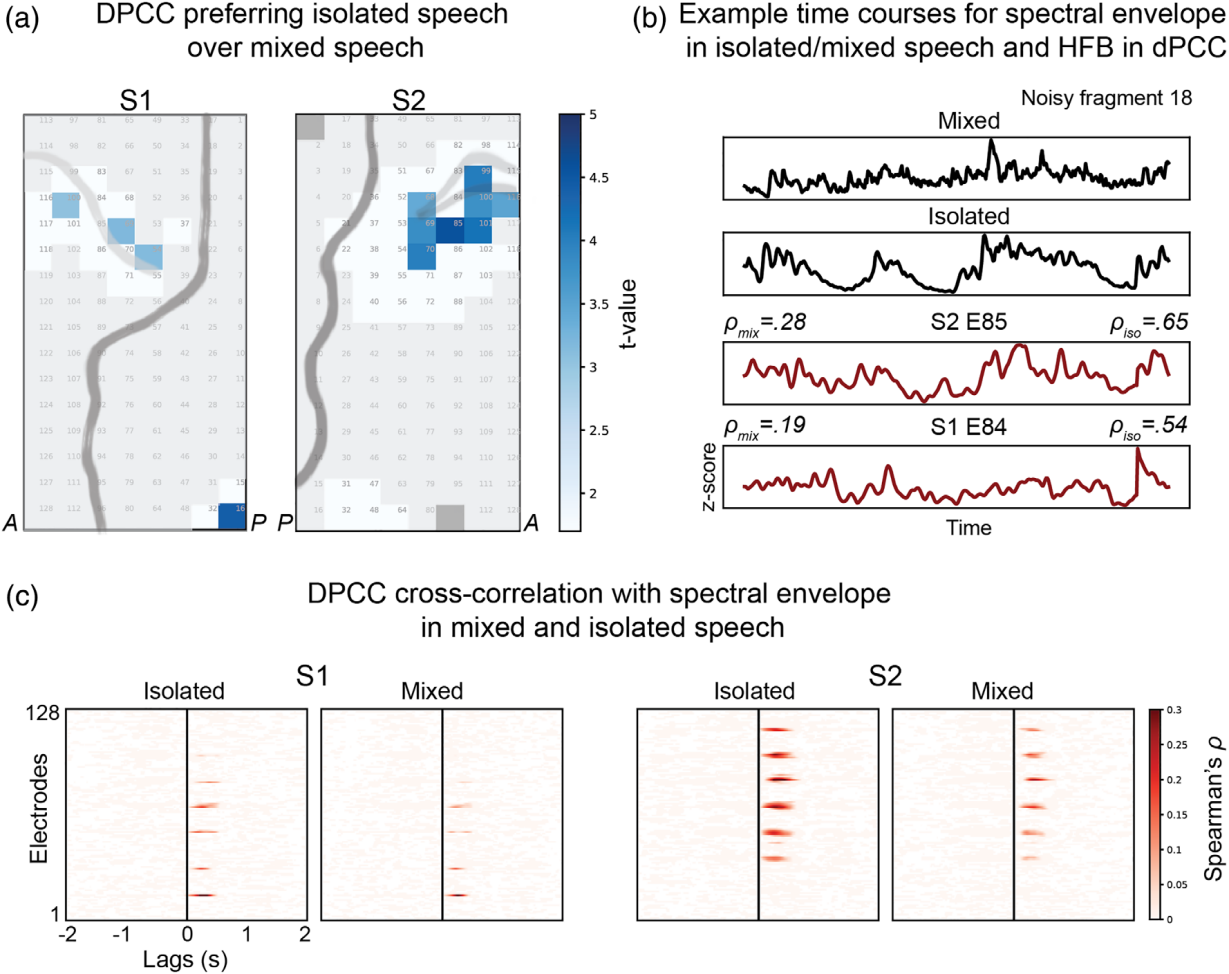
Filtering out of background noise during speech in dPCC. (a) Results of the *t* tests comparing the amount of correlation to the speech spectral envelope in the isolated speech or mixed sound tracks. The correlation and *t* test procedures were identical to the ones previously described, except that in this case the analyses were performed on a different set of fragments that only included noisy speech and speech overlapping with other sounds (see Methods for more details). (b) Example time courses for a noisy speech fragment. From top to bottom: spectral envelope of the fragment in the mixed sound track, spectral envelope of the fragment in the isolated speech track, HFB response of electrode 85 in S2 and HFB response of electrode 84 in S1. (c) Cross‐correlation profiles averaged over all noisy fragments separately for mixed and isolated speech tracks

We obtained separate sound tracks of speech, music and sound effects from the film producer (BosBros Productions, www.bosbros.nl). We selected a new set of speech fragments that contained speech combined with other sounds (*n* = 63). Of note, this set of “noisy” speech fragments was only used in the present analysis and all other analyses were conducted with the previously selected 115 speech and 115 nonspeech fragments. To investigate the effect of background noise, we tested whether dPCC responses to noisy speech fragments were more correlated to the speech spectral envelope of the mixed track (what participants actually heard) or to that of the isolated speech track obtained from the film producer. The *t* test comparing HFB correlation to the speech spectral envelope in both tracks showed that some dPCC electrodes tracked the speech envelope of the isolated track significantly better (${\bar{\rho }}_{S1}=.32\pm .05$ and ${\bar{\rho }}_{S2}=.35\pm .04$) compared with the mixed track (${\bar{\rho }}_{S1}=.28\pm .05$ and ${\bar{\rho }}_{S2}=.29\pm .03$, Figure 3). The reported values show mean *ρ*‐values and *SD* over all significant electrodes (*ρ*‐values were first averaged over all fragments separately for isolated or mixed track per electrode). None of the electrodes showed preference for the mixed track. This result suggests that dPCC was particularly sensitive speech specifically as opposed to the mixed input (speech plus music and noise).

### 3.4 Capturing of the rhythmic phrasal structure of speech in dPCC

The dPCC region preferred isolated speech to the mixed sound track during speech fragments and therefore must have been triggered by speech‐specific properties. One of the auditory properties of speech that is particularly prominent in the absence of background noise is the rhythmic structure of speech. Specifically, when speaking, a continuous stream of speech is typically broken down into phrasal groups by the speaker. These groups are separated by pauses of at least 120–150 ms, but are highly variable in duration (Heldner, 2011; Zvonik & Cummins, 2003). Together, the switches between the groups of speech (phrases) and pauses create a rhythmic phrasal pattern that constitutes one of the key perceptual characteristics of speech.

**FIGURE 4:**
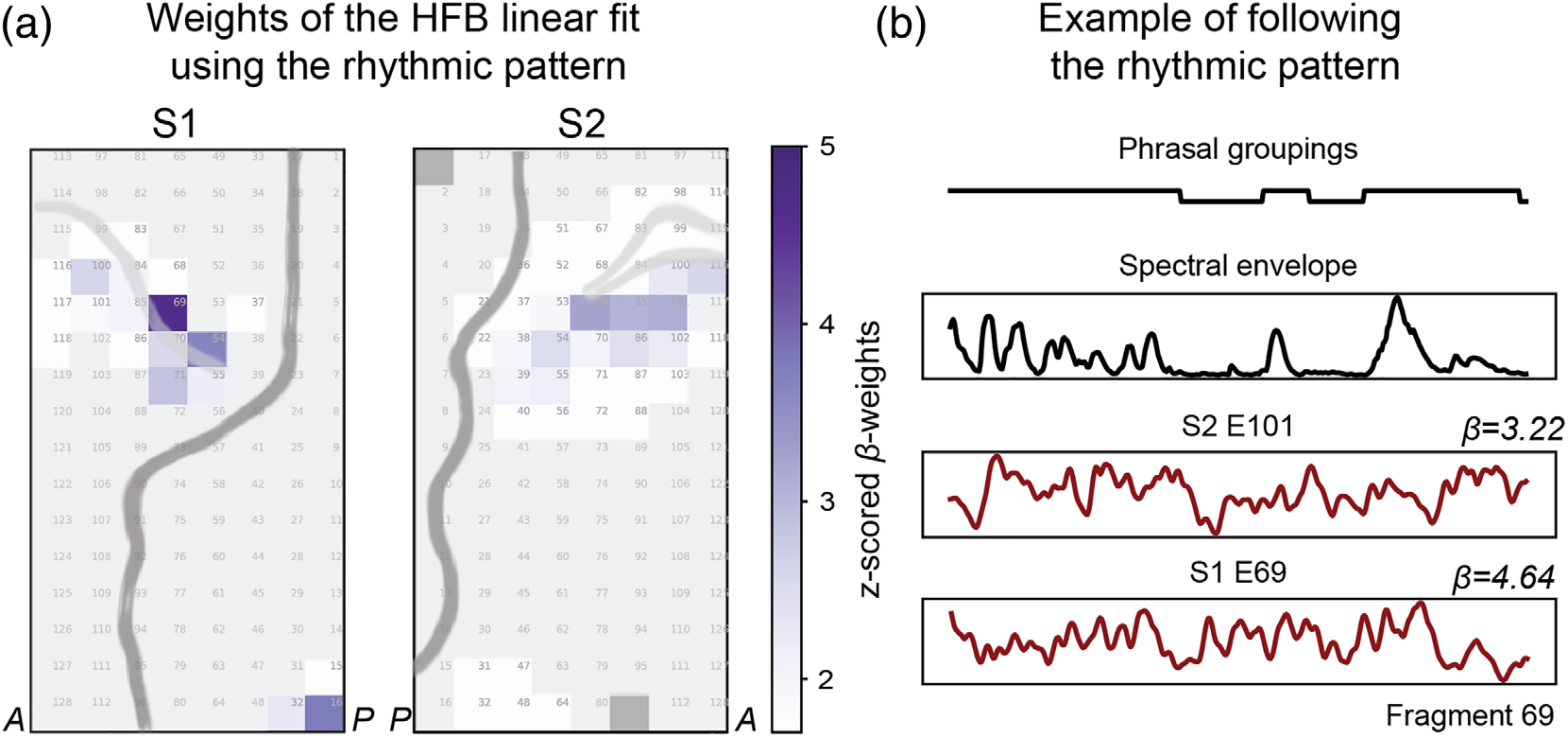
Capturing of the rhythmic phrasal pattern of speech in dPCC. (a) Weights of the linear regression predicting HFB responses based on speech phrasal groupings (ON/OFF speech binary vector). Only speech fragments were used, therefore OFF segments relate to pauses within the continuous stream of speech. (b) Example time courses for a speech fragment showing the rhythmic phrasal pattern in speech (ON/OFF speech binary vector), spectral envelope of the fragment and HFB responses in dPCC in S1 (E69) and S2 (E101). The weight of the linear fit using speech phrasal groupings is also reported per example electrode (*β*)

Following previous indications that dPCC could be involved in tracking of rhythmic properties of speech, including phrasal rates (Keitel et al., 2018), we tested whether in our study dPCC followed the rhythmic phrasal pattern in a continuous stream of speech. For this, we used the previously acquired manual linguistic annotation of the soundtrack (see Methods for details). The annotation contained onsets and offsets of every word in the sound track. Using this information, we constructed a speech ON/OFF binary vector with ones corresponding to speech and zeros corresponding to pauses in a continuous stream of speech. Unlike the spectral envelope analysis, which compared tracking of the spectrotemporal structure in speech and nonspeech fragments, here we focused on the binary structure of the speech input with phrasal groups (coded as 1) delineated by pauses (coded as 0). Thus, only the speech fragments were used (the previously selected 115 speech fragments). We fitted a linear regression to predict dPCC responses to speech fragments using the speech ON/OFF vector. The fit was significant for a large number of electrodes in both S1 and S2: max *F*_S1_ = 2540 and max *F*_S2_ = 3330 (*df*_1_ = 45,998, *df*_2_ = 2), *p* < .001, based on the permutation test (*n* = 10,000). Further inspection revealed a subset of dPCC electrodes with large positive *β*‐weights indicating significant contribution of the speech ON/OFF vector to prediction of HFB responses in those electrodes: ${\bar{t}}_{S1}=33.54\pm 8.57\;$ and ${\bar{t}}_{S2}=36.82\pm 11.14$ (*df* = 45,998) at *p* < .001, Bonf. cor. (Figure 4). For a control analysis, we computed a linear fit using a binary vector of sound being ON/OFF in nonspeech fragments (using a preset sound intensity threshold, see Methods for more details), and compared it against the results of the fit using the speech ON/OFF vector in speech fragments (Figure S4). We found that in both S1 and S2 dPCC electrodes showed considerably more tracking of the ON/OFF sound pattern in speech (its phrasal structure) compared with the general tracking of sound being ON/OFF: *t*_*S*1_(*df* = 38) = 5.22, *p* = 7 × 10^−6^, and *t*_*S*2_(*df* = 80) = 8.12, *p* = 5 × 10^−12^, as tested with independent two‐sample *t* tests. Altogether, these results suggest that a subset of the dPCC electrodes preferentially follow the rhythmic phrasal pattern in a continuous stream of speech.

### 3.5 Capturing of speech pitch in dPCC

Another speech property of high perceptual relevance is pitch. Pitch is associated with the fundamental frequency of the speech signal. Pitch contour encodes changes in intonation of the phrase and voices of individual speakers associated with distinct ranges of pitch magnitude (Bishop & Keating, 2012; Collier, 1975). During speech, pitch is generated by vibrations of the vocal chords and is therefore a characteristic of only the voiced part of the speech signal. At the same time, being a frequency related characteristic of any auditory signal, pitch is not specific to speech and can be extracted from other signals such as music and environmental sounds, for example animal cries (Hevner, 1937; Tramo, Cariani, Koh, Makris, & Braida, 2005).

**FIGURE 5:**
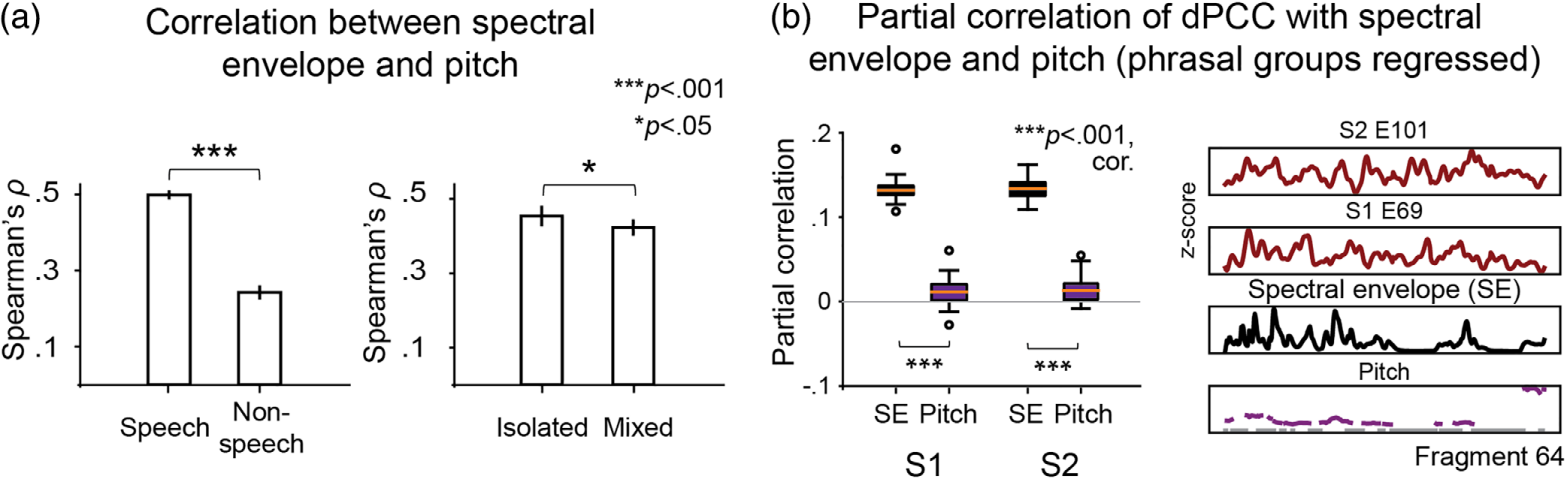
Capturing of speech pitch in dPCC. (a) Spearman correlation between the spectral envelope and pitch. Left panel shows correlation during speech (*n* = 115) and nonspeech (*n* = 115) fragments. Right panel shows correlation during noisy fragments (*n* = 63) in the isolated speech‐only and mixed sound tracks. Error bars indicate the standard error of the mean. The reported difference between conditions (types of fragments on the left or sound tracks on the right) is significant at *p* < .05 or *p* < .001. (b) Partial Spearman correlation of the HFB responses with the spectral envelope and pitch. Data from only the speech fragments were used. To account for the interaction with tracking of the rhythmic phrasal structure, the analysis was performed on residuals of the linear fit of dPCC HFB responses to the binary rhythmic phrasal pattern (see results in Figure 4). Left panel shows the difference in partial correlation with the spectral envelope and pitch for both subjects. Boxes show the 25th and 75th percentiles of the partial correlation values (=correlation computed after having accounted for the third variable: pitch in the case of HFB‐spectral envelope correlation and spectral envelope in the case of HFB‐pitch correlation). Caps show 5th and 95th percentiles. Solid line in the middle shows the median. Right panel shows example time courses of the electrodes with significant partial correlation to the spectral envelope as well as the time course of the spectral envelope and pitch for speech fragment 64

Since pitch contour and the spectral envelope are both related to the frequency component of speech, we first assessed the degree of interaction between them. We found that pitch contour and the spectral envelope correlated significantly during speech fragments ($\bar{\rho }=.5,\;p<.001$) and more than during nonspeech fragments (*t* = 11.01, *df* = 228, *p* = 2 × 10^−22^, Figure 5a). Moreover, the correlation was significantly higher for the isolated speech sound (speech‐only track) compared with speech mixed with noise (mixed sound track, *t* = 2.37, *df* = 61, *p* = .02. On the other hand, both pitch and the spectral envelope inevitably also share information about the rhythmic structure of speech, such that for example during pauses both pitch and spectral envelope have near‐zero values. To account for these interactions and isolate the effects of pitch contour and the spectral envelope tracking, we examined HFB residuals of the previous analysis (regression onto the rhythmic phrasal pattern) and computed their partial correlations with pitch contour (by taking spectral envelope into account) and spectral envelope (by taking pitch contour into account).

The partial correlation analysis showed that dPCC electrodes in both subjects tracked the spectral envelope significantly better (${\bar{\rho }}_{S1}=.14\pm .01$ and ${\bar{\rho }}_{S2}=.14\pm .01$) than pitch contour (${\bar{\rho }}_{S1}=.01\pm .02$ and ${\bar{\rho }}_{S1}=.02\pm .02$) as assessed with paired *t* tests per dPCC electrode: *t*_*S*1_ ranged from 4.11 to 10.23 and *t*_*S*2_ ranged from 4.64 to 9.77 (*df* = 228) at *p* < .01, Bonf. cor. (Figure 5b). This result indicates that the activity of dPCC electrodes was more tightly related to the changes in the spectral envelope rather than pitch contour.

### 3.6 Residual HFB responses to speech and nonspeech fragments

Finally, having observed that dPCC activity reflects various properties of the speech signal, we posed the question of whether the speech properties selected here were sufficient to explain the elevated dPCC response to speech compared with nonspeech fragments. We also assessed if any of these perceptual auditory features could explain the responses of dPCC to nonspeech sounds or whether their tracking was specific to the speech condition only.

**FIGURE 6:**
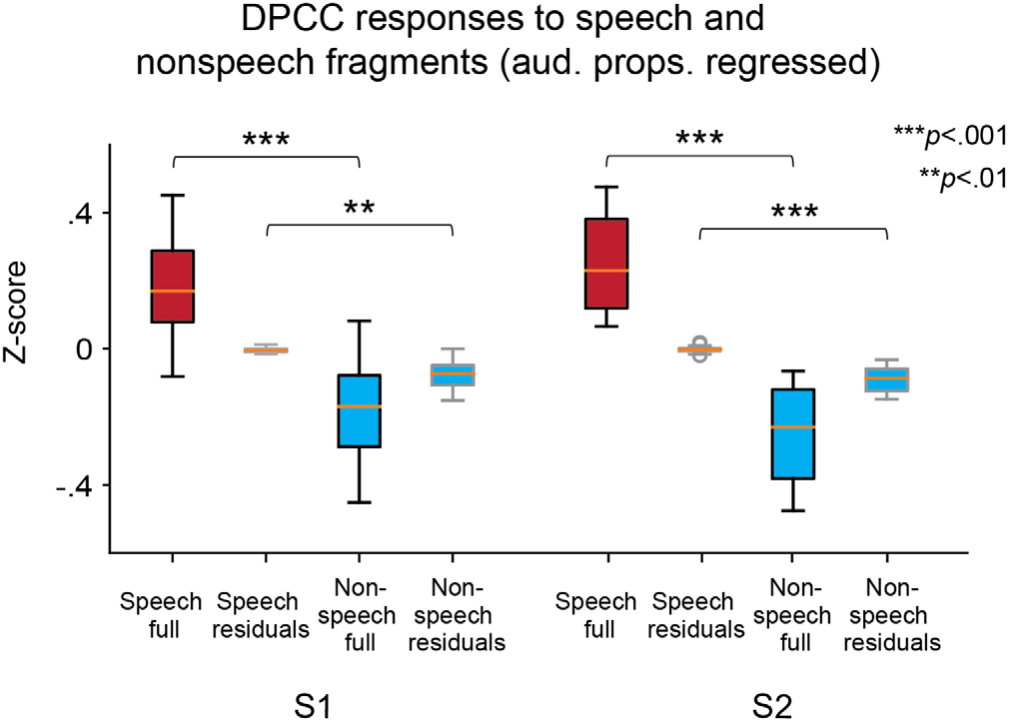
Full and residual HFB responses to speech and nonspeech fragments. Comparison of the average HFB responses to speech (red boxes) and nonspeech (blue boxes) fragments. Original HFB data were used (“full,” boxes with a black outline), as well as the residual HFB data from the regression on all auditory properties (aud. props.): spectral envelope, pitch, rhythmic phrasal structure (boxes with a gray outline). Comparisons between four types of data (“speech‐full,” “speech‐residual,” “nonspeech‐full,” and “nonspeech‐residual”) were performed using a nonparametric version of ANOVA and *t* tests. The reported results are significant at *p* < .01 or *p* < .001. Boxes show the 25th and 75th percentiles of the z‐scored HFB responses. Caps show 5th and 95th percentiles. Solid line in the middle shows the median

For this, we computed a linear fit of the HFB responses in dPCC using the spectral envelope, rhythmic structure (for nonspeech using audio sound being ON or OFF, see Methods for details) and pitch information (*auditory properties*). The fit to the HFB responses was calculated separately for speech and nonspeech fragments. After the fit we obtained residual HFB responses separately for speech and nonspeech conditions. These average residual responses were compared with each other and to the average HFB responses in the original neural data (prior to the linear fit on the auditory properties) using nonparametric alternatives to the standard *t* tests and ANOVA tests (due to the likely violation of the requirement for the equal population variances, see Methods for details). First, we found the effect of the speech condition, once again indicating larger dPCC responses to speech regardless of whether the HFB responses before or after the fit on the auditory properties were used: *F*(1, 76) = 167.57 for S1 and *F*(1, 76) = 741.22 for S2 (Figure 6). Second, the difference between dPCC responses before and after regressing the auditory properties was significantly larger in speech compared with nonspeech (Wilcoxon's *Z*_*S*1_ = 3.57, *p*_*S*1_ = 3 × 10^−4^ and *Z*_*S*2_ = 5.51, *p*_*S*2_ = 4 × 10^−8^). Finally, the normalized amplitude of residual HFB responses to speech was significantly higher than that of the residual HFB responses to nonspeech (Dunn's post‐hoc tests: *m*_speech_residual_ − *m*_nonspeech_residual_ = 0.07, *p* = .008, for S1, and *m*_speech_residual_ − *m*_nonspeech_residual_ = 0.08, *p* = 3 × 10^−5^ for S2). Once again, these results demonstrate that speech perception led to an elevated response in dPCC. Additionally, they indicate that, when put together, auditory properties, such as the spectral envelope, rhythmic structure and pitch contour, explained the dPCC response to speech significantly better compared with the nonspeech input. Moreover, the elevated response of dPCC to speech could not be explained fully by tracking of the auditory properties tested here, as even after regressing these properties out, the dPCC residual response to speech remained elevated compared with its response to the nonspeech input.

### 3.7 Functional specialization in PCC

The previous analyses established a clear connection between the activity within dPCC and speech perception of the feature film. Upon visual inspection, the location of the region appeared to overlap with part of the motor cortex associated with hand movement. To rule out the possibility that the observed effects could be explained by the visual perception of hand movement, we first assessed the interaction between hand movement and speech presence in the film. For this, we annotated all speech and nonspeech fragments with hand presence and hand movement per frame (see Methods for details). Then, we used a *χ*^2^‐test to assess the speech‐hand interaction (Table 2). The test result was not significant, suggesting that there was no interaction between speech and hand conditions in the film data: *χ*^2^(2, 609) = 1.31, *p* = .52.

**TABLE 2:** Contingency table for the *χ*^2^‐test assessing interaction of hand (in video) and speech (in audio) annotations

	Hand movement	Hand presence	No hand	Total
Speech	114	61	107	**282**
No speech	127	62	138	**327**
**Total**	**241**	**123**	**245**	**609**

To determine potential contribution to the brain signals by perceived hand movement in the film, a regression analysis was conducted with hand movement and brain signal in dPCC. The residuals were then entered in the *t* test on the average HFB responses in speech and nonspeech fragments, and the correlation analysis that tested dPCC tracking of the audio spectral envelope. Accounting for the hand movement did not significantly change the previously reported results as tested with two‐sided Wilcoxon signed‐rank tests (*Z*_*S*1_ =  −0.26, *p* = .79; *Z*_*S*2_ =  −0.05, *p* = .96 for the speech preference analysis and *Z*_*S*1_ =  −0.06, *p* = .95; *Z*_*S*2_ =  −0.04, *p* = .97 for the tracking of the spectral envelope analysis). All electrodes remained significant after adding the hand movement covariate.

**FIGURE 7:**
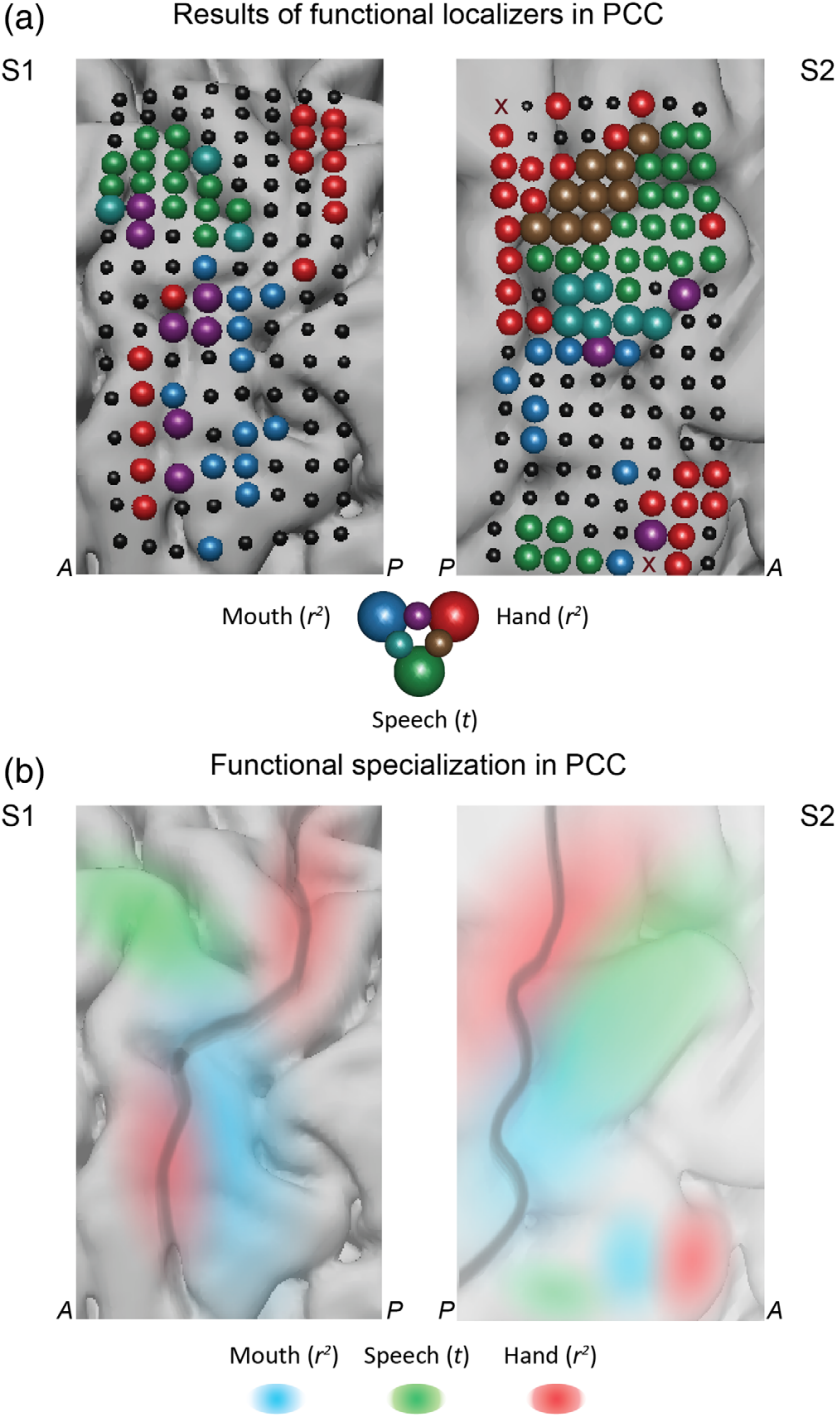
Functional specialization in the precentral cortex. (a) Results of the functional localizers for mapping hand motor (red), mouth motor (blue) and speech perception (green) areas in the sensorimotor cortex. “Hand” localizer was a separate block design task with four conditions: three movement conditions (“thumb,” “index,” “little finger”) and “rest”. “Mouth” localizer was a separate block design task with five conditions: four movement conditions “lips”, “teeth”, “tongue”, “mmmh” (larynx activation) and “rest”. For both localizers (“hand” and “mouth”) the reported results are the r^2^ statistics obtained on the HFB values per electrode when comparing activity during movement conditions and “rest” (see Methods for details). “Speech” localizer is based on the results of the present study and shows comparison of two conditions: speech and nonspeech fragments, also shown in Figure 1b (*t* tests on average HFB values). (b) Schematic representation of the functional specialization in the precentral cortex. Dark gray line outlines the central sulcus. Shading refers to the function of the region: hand motor (red), mouth motor (blue) and speech perception (green)

To assess how the location of speech‐related activity compared with the sensorimotor topography, we used data from separate hand movement and mouth articulation tasks performed by the same patients (see Methods for details). Results are displayed in Figure 7a, showing distinct functional specialization in the sensorimotor cortex with posterior dPCC involved in hand movement, ventral PCC involved in mouth articulation and anterior dPCC involved in speech perception (Figure 7b). Inspection of cortical maps for individual fingers and speech articulators showed that the speech tracking electrodes overlapped most with the larynx articulation map (Figures S2 and S3). Of note, during the laryngeal motor task subjects generated an audible humming sound and it is possible that the activity in dPCC could be related to tracking of the auditory feedback signal.

### 3.8 Reproducibility of results with low‐density ECoG grids

**FIGURE 8:**
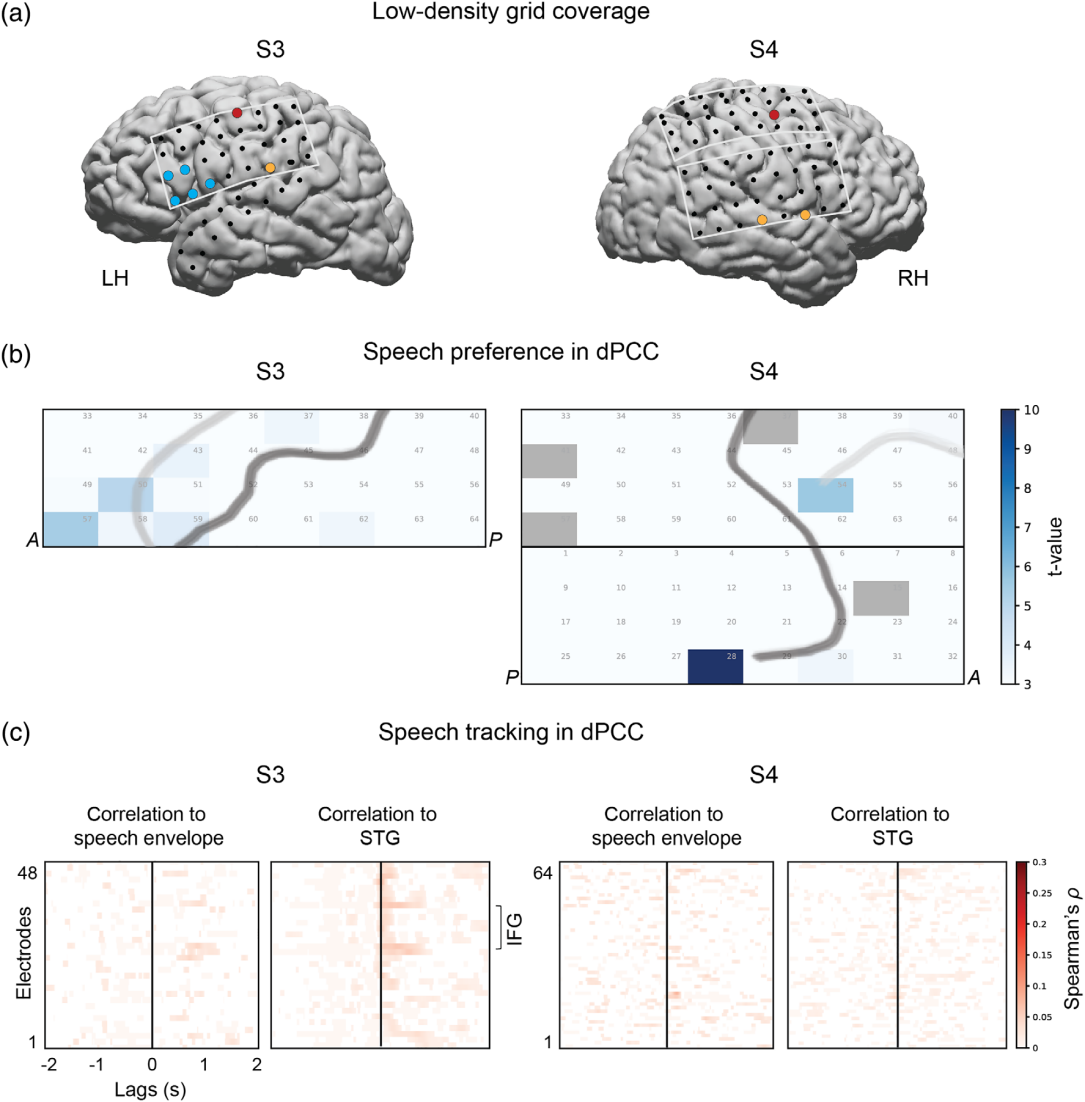
Effects of the grid size. (a) Brain coverage with low‐density clinical grids in two more participants (S3 and S4), who watched the same full‐length feature film. Each dot represents an ECoG electrode. Some electrodes are colored based on their location/function: IFG (blue), STG (orange) and dPCC (red). Only the electrodes with significant *t* values (comparing average HFB in speech and nonspeech fragments) are colored. (b) Results of the *t* test comparing average HFB activity during speech and nonspeech (noise or music) fragments (*p* < .05, Bonferroni corrected for the number of electrodes). (c) Results of speech tracking. The plots show cross‐correlation between HFB and the speech spectral envelope (left panel in both subjects) as well as cross‐correlation between HFB and a STG electrode, which was most correlated to the speech envelope (right panel in both subjects)

Finally, because HD grids are far less common in ECoG research than clinical LD grids (larger diameter and larger inter‐electrode spacing), we sought to confirm some of our results with LD grids. Two participants with LD grids placed over the sensorimotor cortex watched the same film (Figure 8a). We analyzed their HFB responses to the same speech and nonspeech fragments and found a similar tendency for speech preference in anterior dPCC (Figure 8b). Notably, LD grid responses were associated with considerably lower t‐values compared with HD grids: max *t*_S3_ = 3.37 and max *t*_S4_ = 5.65 versus max *t*_S1_ = 27.85 and max *t*_S2_ = 27.74 with only one LD electrode per patient showing a significant effect. The analysis of speech tracking in LD grids (cross‐correlation to speech envelope and STG electrodes) showed no significant results for dPCC (Figure 8c).

## 4 DISCUSSION

In the present study, we investigated and characterized neural responses in PCC to perceived natural speech using HD intracranial recordings. We found that the anterior region within dPCC exhibited preference to perception of speech over other tested sounds. Groups of electrodes within this area displayed tracking of the speech spectral envelope, followed speech phrasal patterns and filtered out background noise. Combining these results with data from additional tasks, we were able to show that this cortical region has a functional specialization distinct from hand motor and mouth articulation functions. Altogether, this work provides evidence that anterior dPCC is actively involved in speech perception. An additional finding was that the response characteristics were less clear in patients with LD intracranial grids, indicating that further research on speech perception (at least in this region) requires the use of HD intracranial electrodes.

### 4.1 Defining dPCC involved in speech perception

**FIGURE 9:**
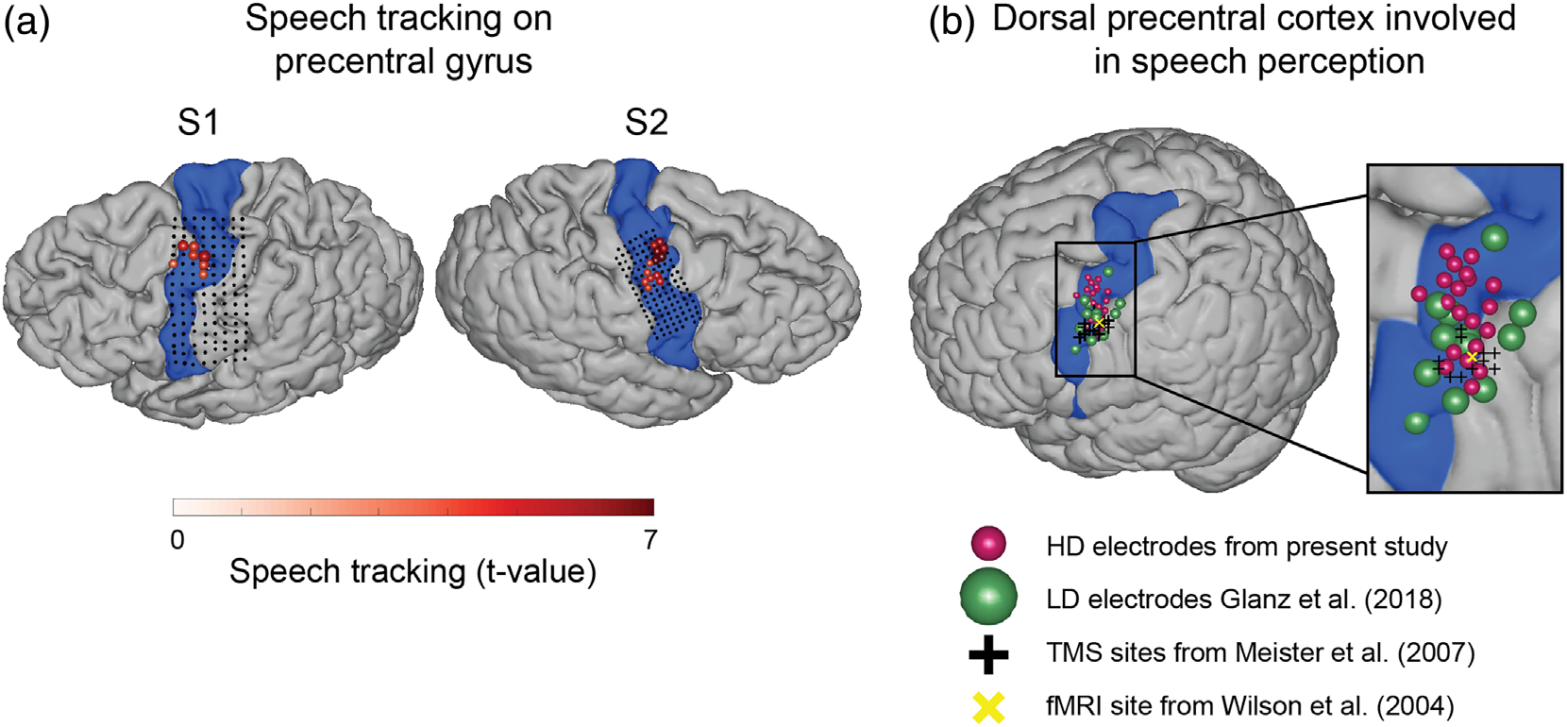
(a) Localization of the speech tracking results in the present study. Colored electrodes show significant tracking of the speech spectral envelope compared with the nonspeech baseline (also shown as a grid plot in Figure 2a). The speech tracking colormap represents *t* values comparing the Fisher‐transformed correlations to the spectral envelope between speech and nonspeech fragments (see Figure 2 and Methods for details). Area corresponding to the precentral gyrus is shown in each participant (highlighted in blue). Anatomical parcellation was performed in the individual subject space using Freesurfer routines. (b) Comparison of the cortical localization of the present results to the literature (on the standard MNI brain). The present results from S2 (right hemisphere coverage) were projected onto the left hemisphere. The MNI coordinates of the present results were obtained using subject‐specific affine transformation matrices computed with SPM8. The results from the previous studies are projected on the standard brain surface using the MNI coordinates reported in those studies. Only left hemisphere coordinates were used. Area corresponding to the precentral gyrus is shown (highlighted in blue). Anatomical parcellation was performed on the MNI brain using Freesurfer routines. Cortical tracking of the speech spectral envelope from (a) is shown (electrodes in red). Electrodes that show response to both perception and production of speech during real‐world conversations from Glanz et al. (2018) are displayed in green. Center of the area responding to perceived syllables as measured with fMRI and reported in Wilson et al. (2004) is shown (yellow x). Finally, sites, whose stimulation with TMS affected perception of consonants reported in Meister et al. (2007) are displayed as black crosses (one per individual subject)

The present findings provide strong evidence of the involvement of anterior dPCC in speech perception (Figure 9a). Previous research has implicated involvement of similarly located or neighboring regions during perception of speech with functional magnetic resonance imaging (fMRI; Du et al., 2014; Skipper et al., 2005), magnetoencephalography (Keitel et al., 2018), low‐density ECoG (Cogan et al., 2014; Ding, Melloni, Zhang, Tian, & Poeppel, 2016; Glanz et al., 2018) and transcranial magnetic stimulation (TMS; Floel et al., 2003; Meister et al., 2007). However, the exact location and therefore functional specificity of this region remain undefined. Various studies consider the region to be part of the premotor cortex rather than motor cortex (Glanz et al., 2018; Meister et al., 2007), even though some of the reported coordinates seem to belong to motor cortex proper according to the boundary delineated in a large meta‐analysis study (Mayka, Corcos, Leurgans, & Vaillancourt, 2006). Some researchers point out that the observed effect is at the border of premotor and motor regions (Wilson et al., 2004). Inconsistency extends to defining the boundaries of dorsal and ventral cortices as well (Mayka et al., 2006). Several studies refer to the region as part of the dorsal (pre)motor cortex (Keitel et al., 2018; Meister et al., 2007), whereas others call it superior part of the ventral (pre)motor cortex (Cheung et al., 2016; de Heer, Huth, Griffiths, Gallant, & Theunissen, 2017; Glanz et al., 2018; Wilson et al., 2004). The boundary also differs between meta‐analyses of neuroimaging and cytoarchitectonic data (Mayka et al., 2006; Rizzolatti & Luppino, 2001). The lack of distinct anatomical definition combined with a considerable variability in localization across individuals (Glanz et al., 2018) mark the challenge in delineating functional topography.

Despite the difference in terminology, neural recording modalities, and experimental paradigms, the present results show a considerable overlap in location with several previous reports (Figure 9b). Wilson et al. (2004) used fMRI to locate a site in dPCC that responds to perception of syllables. A recent study by Glanz et al. (2018) combined LD ECoG and electrical stimulation mapping (ESM) to identify a region in superior ventral premotor cortex involved in both production and perception of naturalistic speech. Somewhat similar to the LD results reported here (Figure 8a), Glanz et al. (2018) showed that only a few LD electrodes (eight across 12 patients) in dPCC responded to naturalistic speech.

The present study differs from these two and the previously mentioned reports in several ways. First, we take advantage of HD neural recordings to obtain a detailed map of the function in dPCC. Second, we examined neural activity while participants were watching a feature film, eliminating constraints induced by a specific cognitive task. This is in contrast to Glanz et al. (2018) who also used a naturalistic experimental setup but analyzed speech perception and production moments in real‐world conversations. Thus, our results cannot be attributed to motor planning or predictions as part of a face‐to‐face interaction and are due to speech perception proper. This approach made it possible to associate a distinct portion of dPCC with a processing of features that are specific to speech perception. The specificity revealed by tracking of the spectral envelope and varying phrasal structure of naturalistic speech has, to the best of our knowledge, not been reported before.

### 4.2 Relation to hand and mouth motor processes in dPCC

The present results show reliable activation of anterior dPCC by perceived speech. However, given previous research and the region's location in the brain, it is important to consider our findings in the context of motor processing. In particular, the mirror neuron theory implicates neurons in (pre)motor cortex in both perception and execution of goal‐oriented action, particularly emphasizing their role in action understanding (Di Pellegrino, Fadiga, Fogassi, Gallese, & Rizzolatti, 1992; Rizzolatti & Craighero, 2004). Even though the mirror neuron theory has met considerable criticism (Hickok, 2009), many researchers continue to agree that the observed neural activity of the (pre)motor region in both human and nonhuman primates reflects some form of interpretation of the perceived actions (Salo, Ferrari, & Fox, 2019).

The dPCC is primarily associated with hand motor processing, and one could argue that the present results could be attributed to the fact that the motor cortex merely responds to perceived communicative hand gestures. We find this explanation unlikely for several reasons. First, the present results rely on correlations of the dPCC HFB activity to the speech spectral envelope, which captures the slowly varying shape of the speech signal. The spectral envelope has been shown to be critical for perception of individual phonemes as well as overall sentence comprehension (Arai et al., 1996; Ter Keurs et al., 1993). Many core regions involved in speech processing show tracking of this speech feature (Kubanek et al., 2013). Second, accounting for hand presence and movement in the film frames did not change the present results in any of the electrodes, suggesting that anterior dPCC is unlikely to respond to perceived actions and hand movements, but is rather related to tracking of speech‐specific information. Finally, utilizing high spatial resolution of HD electrodes we were able to map individual hand movements on dPCC, and found their location to be different from the area that tracked perceived speech (Figures 7 and 8a).

The notion of the motor cortex supporting both action perception and execution is at the core of the motor theory of speech perception. It posits that the cortical regions implicated in mouth articulation (ventral PCC, “face area”) and motor planning (ventral premotor cortex) could subserve simulation and phonological prediction processes during speech perception (Cheung et al., 2016; Pulvermüller et al., 2006; Skipper et al., 2007). However, the region found in our study is located considerably more superior to the mouth motor region in both subjects, indicating that it is separate from ventral PCC proper (Figures 7 and S3). At the same time, there appeared to be a considerable overlap with the dorsal laryngeal motor region identified in this study (Figure S3), which has recently been reported to subserve volitional control of pitch (Dichter et al., 2018). Here, we show that the identified region tracked properties in perceived speech beyond pitch (Figure 5b). Moreover, regressing various acoustic features from the neural responses (including pitch contour) did not account entirely for the dPCC elevated response to speech compared with nonspeech sounds (Figure 6). Altogether, this evidence suggests that either the identified dPCC region has other function in addition to laryngeal motor control or the currently considered laryngeal function of dPCC should be revised. Of note, it is currently considered that there are two laryngeal motor regions: one in dPCC and another one in ventral PCC (Bouchard et al., 2013; Brown et al., 2007; Simonyan & Horwitz, 2011). Only the dorsal region tracks perceived speech in our study.

In addition, one of the HD ECoG subjects of the present study (S2) showed an elevated response to speech in the ventral PCC proper (Figures 1b and S4). These electrodes were included in all further analyses (as part of the electrode mask, see Figure 1b), yet we did not observe consistent tracking of the spectral envelope of speech in that region. This appears to be in contrast with results reported by Cheung et al. (2016), who showed neural tuning of the ventral motor cortex to acoustic properties of speech. Importantly though, Cheung et al. (2016) reported that there were two groups of sensorimotor electrodes that responded to perceived speech: one in the inferior and another in the superior ventral somatosensory cortex. Considering our previous discussion of the terminology, we believe that their superior ventral sensorimotor cortex may overlap with what we refer to as dorsal PCC here. Interestingly, the cortical maps in Cheung et al. (2016) for the HFB response to perceived speech and the neural fit using acoustic features suggest a possibly larger involvement of superior ventral sensorimotor (or dorsal PCC) electrodes in tracking of speech compared with the inferior ones, that appear to be in the classical “face area.” Those results were obtained from a controlled task, where patients listened to isolated syllable sequences, and it is possible that using a long‐duration naturalistic stimulus in our study further reinforces this effect. More research focusing on the differences in the response profiles between these two areas on the motor cortex is undoubtedly needed to advance our understanding of the motor cortex response to perceived speech.

### 4.3 The role of dPCC in speech perception

The finding of a distinct region, just anterior to the “hand knob” and superior to the “face area,” that tracks auditory properties of speech raises questions about its function. Several ideas about the function of this region have been previously reported, including generation of forward motor representations of speech sound, facilitatory mechanisms for perception under difficult conditions, and a role in prediction and processing of temporal information in speech.

Meister et al. (2007) reported that repetitive TMS stimulation of the premotor region (dorsal and anterior to the central sulcus) leads to a significant decline in subjects' ability to discriminate consonant sounds presented in noisy conditions. The authors suggested that this area is crucial for mapping of acoustic representations of speech sounds onto corresponding articulatory gestures. They theorized that premotor cortex might feed these top‐down motor representations forward to STG for their comparison against the acoustic input and thus have a causal role in speech perception. In the present study, we find consistent involvement of the same region (dorsal and anterior to the central sulcus) in tracking of perceived continuous speech. At the same time, we do not find support for the notion of feedforward processing from dPCC to STG given that we do not observe any consistent lag between HFB activity in dPCC and STG that one would expect in a feed‐forward theory.

An alternative theory explaining activation of (pre)motor cortex during speech perception is based on the hypothesis of its facilitatory rather than causal function during perception of speech. This theory is based on activation of (pre)motor cortex during perception of noisy and degraded speech (Callan et al., 2003; Callan, Jones, Callan, & Akahane‐Yamada, 2004; Du et al., 2014). The facilitation effect is thought to be achieved through sensorimotor integration and engagement of an internal model that maps speech sounds to articulation. Although the effect was reported to be localized to the ventral premotor cortex (Du et al. (2014), the speech perception maps for both noisy and clean speech seem to include dPCC as well. Sato, Tremblay, and Gracco (2009) suggested that the facilitatory function of the (pre)motor region should manifest in one of two scenarios: (a) when varying task complexity (Du et al., 2014; Wilson & Iacoboni, 2006) or (b) during conversational exchange (Foti & Roberts, 2016; Scott, McGettigan, & Eisner, 2009). Neither option can fully account for the activation of this region during passive perception of a narrative, such as a feature film. It is possible that the multisensory integration during the audiovisual perception can contribute to the present results. However, this would mean that various reports of dPCC involvement are due to different conditions: noise and task difficulty (Meister et al., 2007; Sato et al., 2009), conversation exchange (Glanz et al., 2018), and passive listening to naturalistic speech (present work). We tend to consider this unlikely and instead believe that dPCC simply elicits a more basic, fundamental response to perceived speech.

A different line of work associates the activation in dPCC with cortical entrainment to rhythmic features of individual sentences (Bengtsson et al., 2009; Ding et al., 2016; Keitel et al., 2018). In this view, dPCC involvement in speech perception ultimately connects to temporal prediction and tracking of rhythmic structure in (pre)motor cortex (Chen, Zatorre, & Penhune, 2006; Morillon, Schroeder, & Wyart, 2014). We find that anterior dPCC follows phrasal grouping patterns in the continuous stream of speech. At the same time, regressing out the temporal acoustic properties from HFB responses does not entirely remove the difference in activity associated with perception of speech compared with nonspeech sounds (Figure 6). Moreover, there appears to be a clear encoding of the frequency components of speech (spectral envelope and pitch contour). This indicates capturing of information beyond the rhythmic structure in dPCC and aligns with results from Meister et al. (2007) where inhibition of dPCC with repetitive TSM affects the subjects' ability to discriminate between perceived consonants—a task that is free from any temporal pattern.

In sum, findings in literature as of yet do not provide a comprehensive account for the function of dorsal (pre)motor/precentral cortex in perception of speech. Apart from theories embedding the function of dPCC in some form of motor processing, other reports interpret dPCC responses to speech in terms of semantic (de Heer et al., 2017), lexical (Duffau et al., 2003), and verbal memory (Müsch, Himberger, Tan, Valiante, & Honey, 2020) processing. A more unifying theory of the function of this region that could explain its involvement in both perception and production of speech (Glanz et al., 2018; Wilson et al., 2004) remains much needed in the field. We believe our current findings provide some direction to this endeavor.

With the spatial and temporal resolution of recordings in the current study, a close association between a distinct dPCC region and processing of perceived speech was observed. To better understand the functional relevance of this, further investigation of functional sequelae following virtual lesioning in this area seems warranted, for instance with ESM over HD grid electrodes. Less specific virtual lesion techniques such as TMS have elucidated some of the relationship between speech perception, speech production and hand motor cortices (see a review Möttönen & Watkins, 2012). An overlap in speech production and perception was observed by stimulating through standard LD electrode grids in dPCC (Glanz et al. (2018). This study, however, also reported motor effects spanning multiple body parts including lips, tongue, neck, eyes, chin, head, and fingers, suggesting perhaps a lack of specificity of stimulation. Only the study by Dichter et al. (2018) reports on ESM over HD grid electrodes, where a topographically similar region displayed motor effects on the larynx (other articulators were not tested). A study on patients with lesions in premotor cortex showed that electrical stimulation of dPCC led to no articulation deficits but rather difficulties in object naming (Duffau et al., 2003). Given that the spatial resolution of TMS and standard LD ECoG is in the range of 1 cm (Roth & Hallett, 1992; Thielscher & Kammer, 2002), it may well be that separation and in‐depth investigation of functions in the regions we report on, requires more specific stimulation (HD grid). Given our findings, a focus on separating motor and language functionality would be of interest.

### 4.4 Addressing the possibility of acoustic contamination in the ECoG signal

A recent report (Roussel et al., 2019) raised a possibility that audio signals may affect the integrity of ECoG data, because of a specific wiring setup and injection of mechanically‐induced electrical noise. Heeding this report, we examined our results in this light. We did not find evidence for presence of a mechanical–electrical artifact, given the observations that (a) the ECoG‐to‐audio correlation we report showed a lot of variability across different parts of the film, which would not be expected if acoustic waves (present throughout the movie) were driving ECoG signals; (b) ECoG‐to‐audio correlations were only significant at a temporal lag of up to 300 ms (Figures 2a and S5); (c) the effects we report are present at a lower frequency range than the 115 Hz and up that Roussel et al. (2019) report (recalculated and shown in Figure S5).

### 4.5 Limitations and future work

The present work has a number of limitations. For one, due to the rarity of HD recordings, data from only two HD participants were available. Complimentary work with low‐density grids suggests that HD recordings are necessary for accurate mapping of function in dPCC.

Second, it is possible that the present results were confounded by perception of the visual stream of the film and particularly by perception of movement. By labeling hand presence and movements in the film, we could correct for the latter confound to some degree. Analyses of the data after removing any hand presence or movement did not affect the statistical results, suggesting that the confound was minimal at best.

Related to this, it is possible that eye movements could have interfered in comparing brain activity during speech and nonspeech fragments. However, it is unlikely for eye movements to correlate with various auditory properties of speech including frequency‐based characteristics such as the spectral envelope and pitch. It may be interesting to expand the present work by analyzing the electrooculography recordings and investigate saccade movements with respect to processing of the visual component of speech fragments and possible contribution of attentional mechanisms.

Finally, the present results were only limited to the HFB component of the neural signal. This was due to the fact the HFB activity closely corresponds to the local neural firing rates (Crone et al., 1998; Ray et al., 2008), on the one hand, and matches well the blood‐oxygenation‐level‐dependent response (Hermes et al., 2012; Lachaux et al., 2007), on the other hand. In addition, we were able to show that using the exceptional spatial resolution of the HD ECoG grids we could recover local neural behavior that was nearly undetectable with low‐density ECoG grids. However, other components of the neural signal (lower frequency bands) as well as cross‐frequency coupling have also been shown to play an important role during speech perception (Assaneo & Poeppel, 2018; Ding et al., 2016; Giraud & Poeppel, 2012; Keitel et al., 2018; Park, Ince, Schyns, Thut, & Gross, 2015), and thus constitute one of the main directions for future work with these data.

Conceptually, this work could be complemented in a number of ways. For example, further analyses of the difference in the responses of the ventral and dorsal PCC could clarify the distinctive functions of these subregions of the motor cortex in speech perception. Another promising extension is the in‐depth analysis of the connectivity between motor and auditory regions. The cross‐correlation results shown here indicate parallel coactivation of the two regions. This effect has been reported before along with the existence of other neural populations in the motor cortex whose activation either precedes or follows STG (Cheung et al., 2016). More work is required to clarify how the sensorimotor circuit is activated by incoming speech sound and what role the STG‐motor connectivity plays in perception of speech.

## 5 CONCLUSIONS

We investigated the involvement of precentral cortex (PCC) in continuous speech perception using high‐density (HD) intracranial recordings. Our results show that a specific region within the dorsal portion of PCC (dPCC) tracks various properties of speech including, but not limited to, its spectral envelope, pitch contour, and rhythmic phrasal groupings even with additional background noise or sounds. Tracking occurs in parallel to the activity in the superior temporal cortex. The location of the identified region is distinct from the hand motor and mouth articulator areas. In addition, we find that these results are more pronounced in HD grid participants compared with standard intracranial grids, indicating the importance of both spatial and temporal detail in studying neural responses to speech perception on the sensorimotor cortex.

## CONFLICT OF INTEREST

The authors declare no conflicts of interest.

## Supporting information


**Appendix** S1: Supporting information

## Data Availability

The data supporting the current study have not been deposited in a public repository due to the restrictions on public sharing of the patients’ data but are available on request. Interested parties can contact us at http://www.nick‐ramsey.eu/contact/. The code used to obtain the reported results is available at https://github.com/Immiora/tracking_speech_adpcc_ecog_hd.
