# Supplementary material for: High‐density intracranial recordings reveal a distinct site in anterior dorsal precentral cortex that tracks perceived speech
Source: Hum Brain Mapp. 2020 Aug 3;41(16):4587–609. doi: 10.1002/hbm.25144 (PMC7555065; doi:10.1002/hbm.25144)
Supplement: Supplementary file 1 — Appendix S1: Supporting information [file HBM-41-4587-s001.docx]

University Medical Center Utrecht

High-density intracranial recordings reveal a distinct site in anterior dorsal precentral cortex that tracks perceived speech

Dorsal precentral cortex tracks continuous speech

**SUPPLEMENTARY MATERIAL**

Julia Berezutskaya¹ ², Clarissa Baratin¹, Zachary V. Freudenburg¹ and Nicolas F. Ramsey¹

Brain Center, Department of Neurology and Neurosurgery, University Medical Center Utrecht, Heidelberglaan 100, 3584 CX,Utrecht, The Netherlands¹

Donders Institute for Brain, Cognition and Behaviour, Radboud University, Montessorilaan 3, 6525 HR, Nijmegen, The Netherlands²

**Supplementary table 1.** MNI-coordinates of the HD electrodes with significant tracking of the speech spectral envelope against the non-speech baseline (Figures 2a and 6a).

| **Area** | **MNI coordinate** | | | **Patient** |
| --- | --- | --- | --- | --- |
|  | **x** | **y** | **z** |  |
| Left precentral gyrus | -46 | -10 | 61 | S1 |
| Left precentral gyrus | -48 | -9 | 58 | S1 |
| Left precentral gyrus | -51 | -9 | 55 | S1 |
| Left precentral gyrus | -41 | -6 | 63 | S1 |
| Left precentral gyrus | -44 | -5 | 61 | S1 |
| Left precentral gyrus | -39 | -2 | 63 | S1 |
| Left precentral gyrus | -45 | 4 | 57 | S1 |
| Right precentral gyrus | 57 | -7 | 51 | S2 |
| Right precentral gyrus | 59 | -6 | 49 | S2 |
| Right precentral gyrus | 54 | -7 | 54 | S2 |
| Right precentral gyrus | 56 | -5 | 52 | S2 |
| Right precentral gyrus | 51 | -6 | 57 | S2 |
| Right precentral gyrus | 54 | -3 | 53 | S2 |
| Right precentral gyrus | 56 | -1 | 50 | S2 |
| Right precentral gyrus | 50 | -4 | 58 | S2 |
| Right precentral gyrus | 44 | -5 | 62 | S2 |
| Right precentral gyrus | 46 | -4 | 60 | S2 |
| Right precentral gyrus | 48 | -2 | 59 | S2 |
| Right precentral gyrus | 42 | -3 | 63 | S2 |
| Right precentral gyrus | 44 | -1 | 61 | S2 |


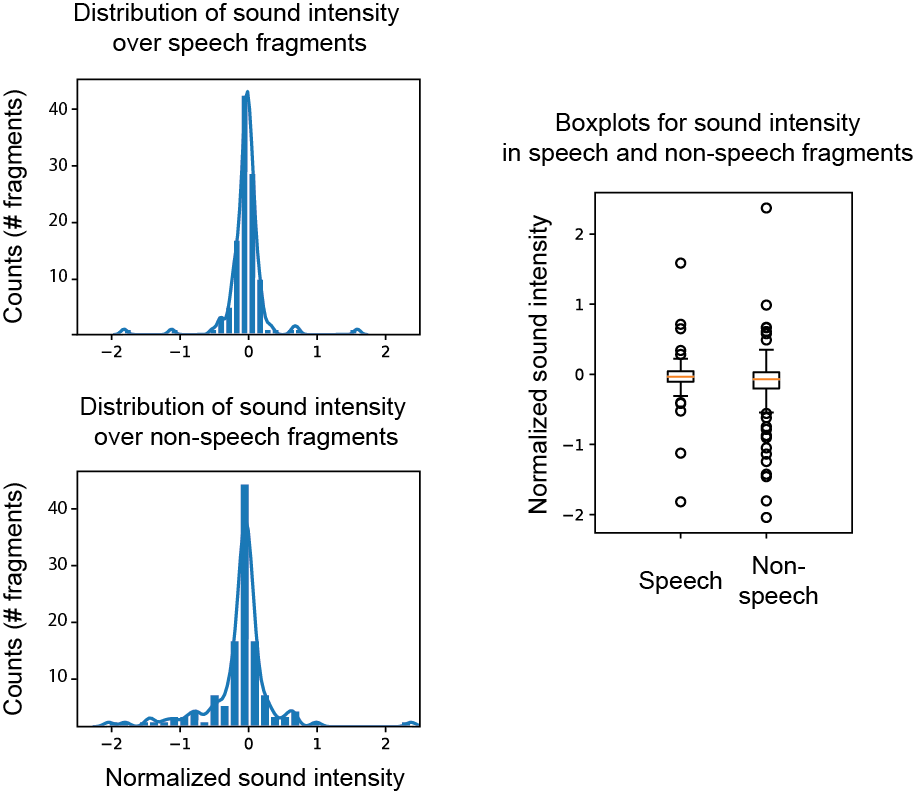


**Supplementary figure 1.** Control for sound intensity in speech and non-speech fragments. Panels on the left show histograms of normalized sound intensity (normalized over the entire soundtrack) for the speech and non-speech fragments separately. Panel on the right shows boxplots of the normalized intensity separately per condition (speech and non-speech fragments). Boxes show the 25^th^ and 75^th^ percentiles of the sound intensity values, caps show 5^th^ and 95^th^ percentiles. Circles represent the outliers. Solid line in the middle shows the median.


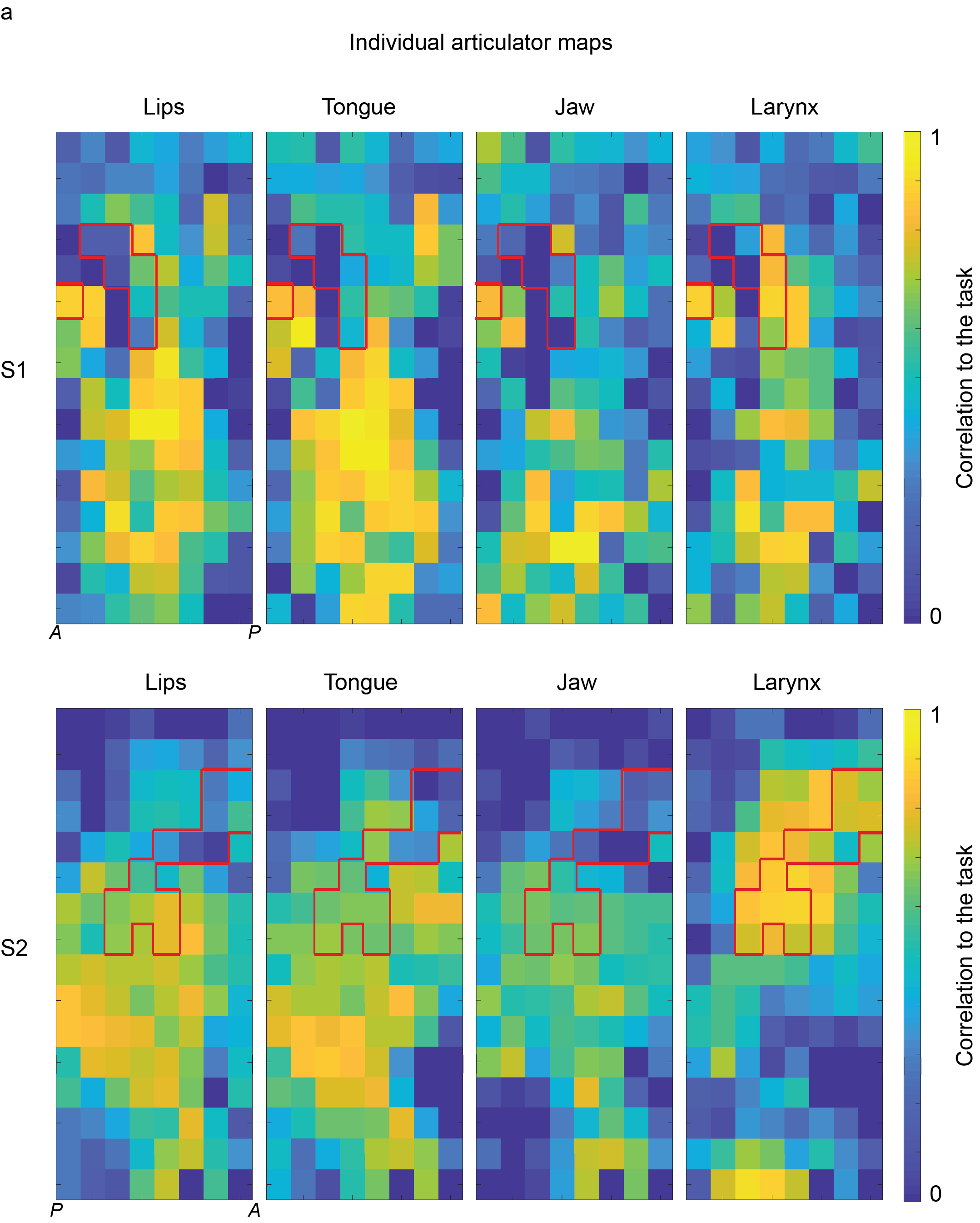


**Supplementary figure 2.** Cortical maps (electrode grid plots) for individual speech articulators: lips, tongue, jaw and larynx. Red contour outlines electrodes involved in speech tracking (**Figures 2a** and **9a** of the main text).


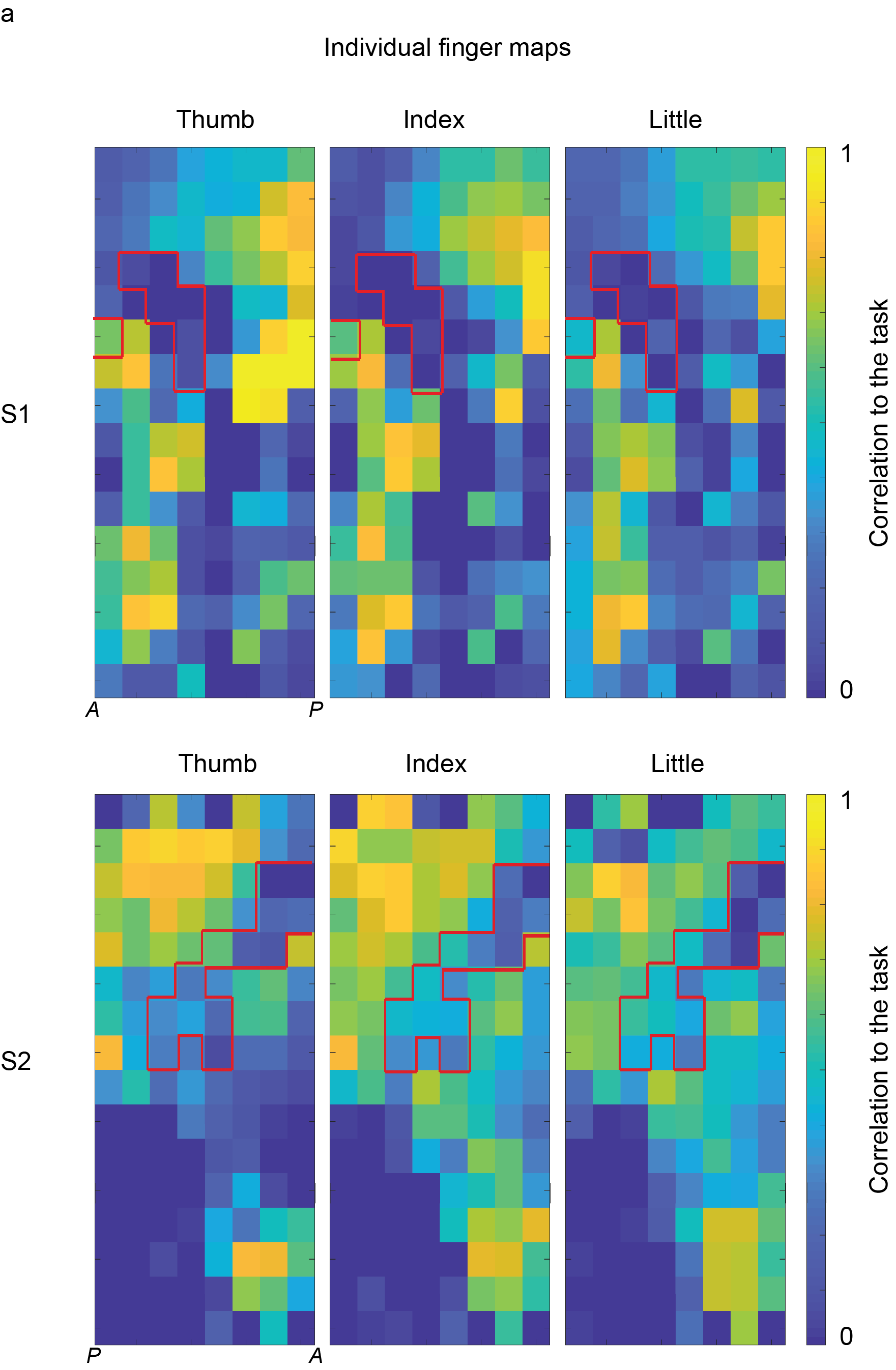


**Supplementary figure 3.** Cortical maps (electrode grid plots) for individual finger movements: thumb, index and little finger. Red contour outlines electrodes involved in speech tracking (**Figures 2a** and **9a** of the main text)

**
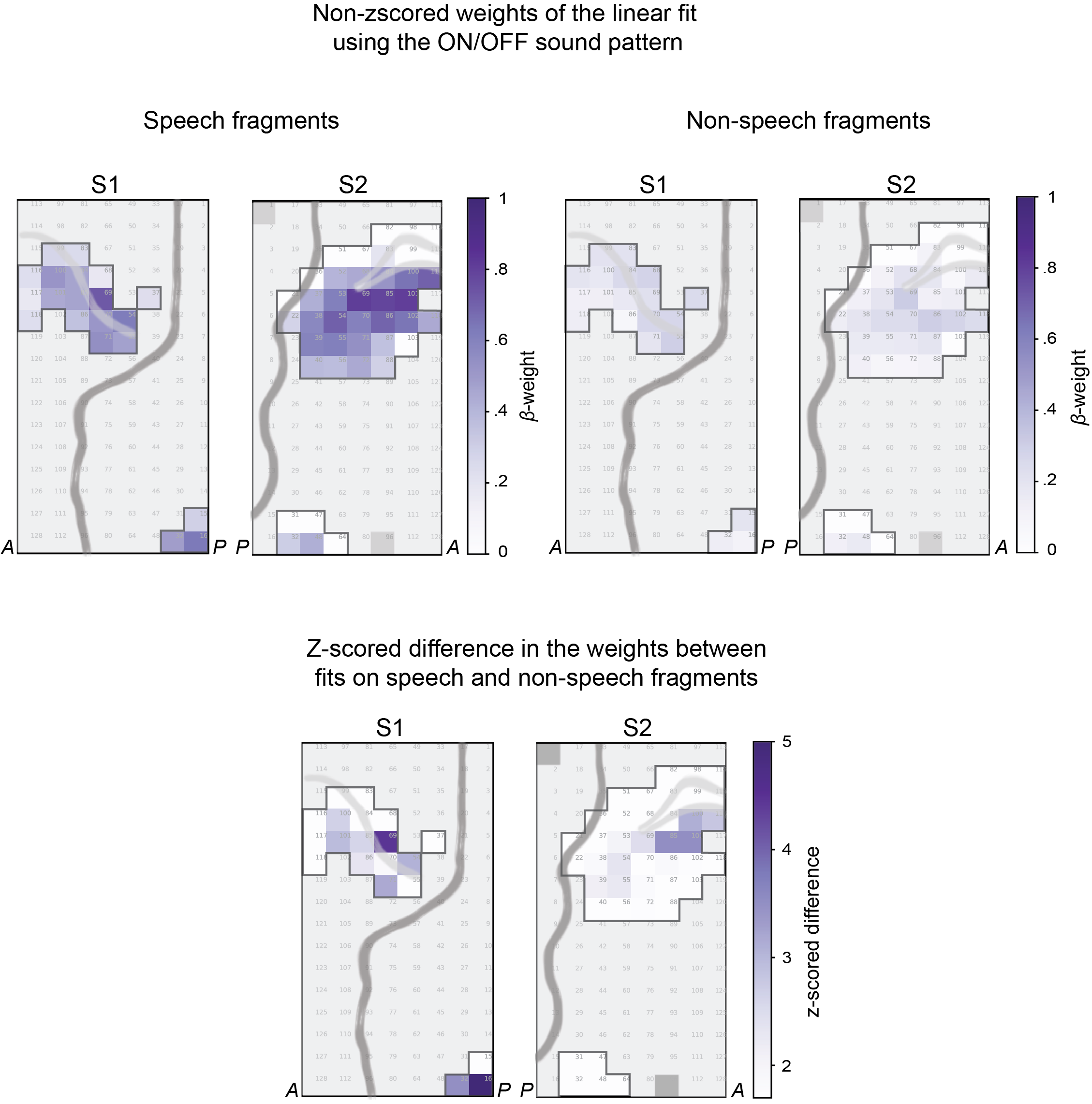
**

**Supplementary figure 4.** Results of the control analysis for capturing of the sound ON/OFF patterns in dPCC. Top left panel shows non-zscored weights of a linear regression predicting HFB responses based on speech phrasal groupings (ON/OFF speech binary vector). Zscored weight maps from this analysis are reported in the main text (**Figure 4**). Top right panel shows the results of the analogous analysis during non-speech fragments. Specifically, it shows non-zscored weights of a linear regression predicting HFB responses during non-speech fragments based on the sound ON/OFF binary vector (using a fixed sound intensity threshold, see Methods for details). Bottom panel shows a zscored difference between the weight maps above highlighting a stronger preference in the dPCC electrodes to follow the speech phrasal structure compared to a simple sound ON/OFF pattern.

**
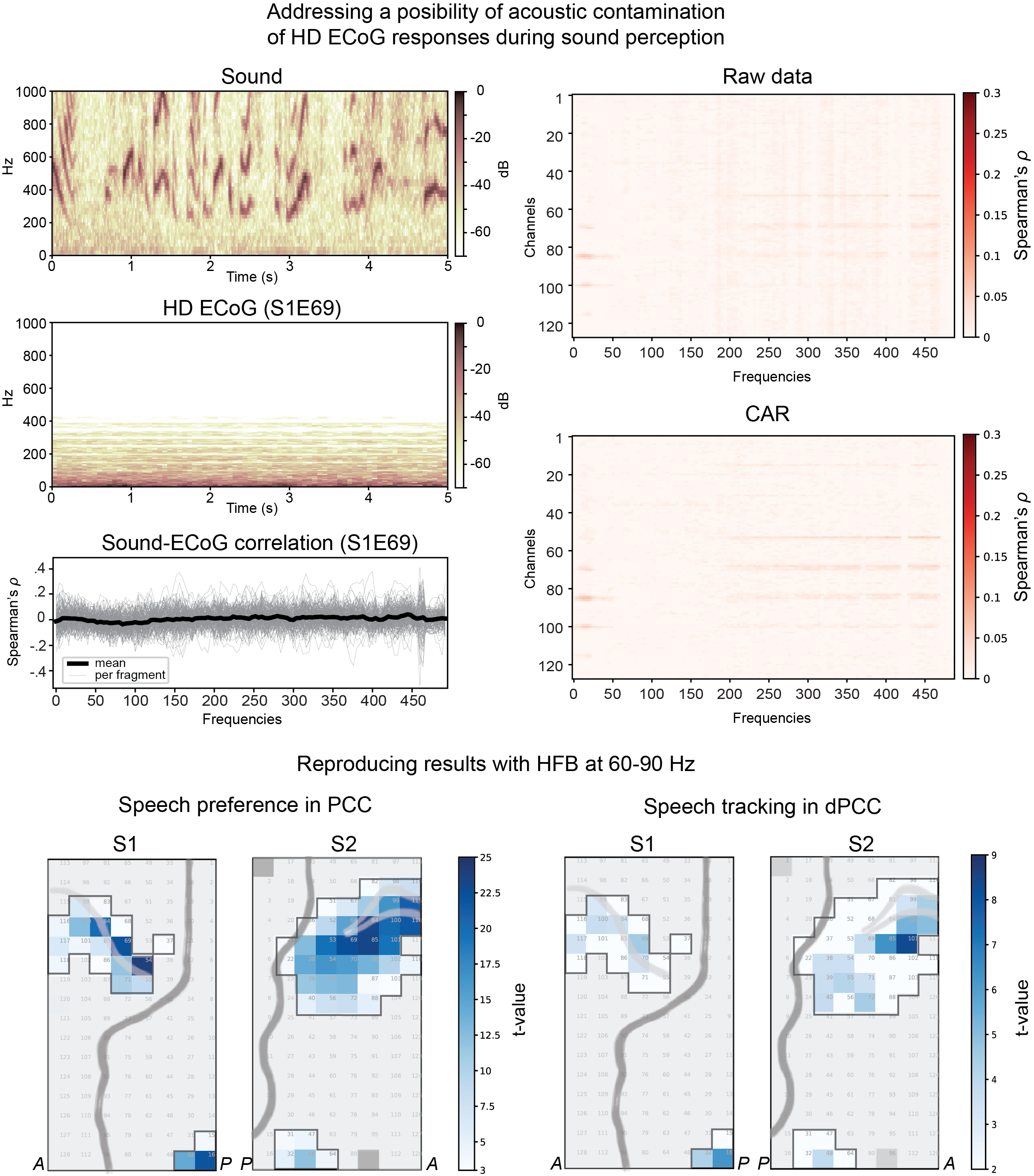
**

**Supplementary figure 5.** Addressing a possibility of acoustic contamination of HD ECoG responses during sound perception. Top left panels show example spectrograms of the film soundtrack and HD ECoG signals. The ECoG signal of S1, electrode 69 was used. This subject watched the entire film in one go, which means that any variation in the audio-to-ECoG correlation is due to the nature of the neural response and not due to any possible changes in the recoding setup, such as a change of the reference electrode, wiring adjustments, audio volume, etc. The chosen electrode showed a strong response to speech in various types of analysis. The spectrograms were obtained using the *librosa* (<https://librosa.github.io/librosa/>) package. It calculated the shot-time Fourier transform of the signal with a window length of 46 ms for audio (at the sampling rate of 22050 Hz) and 256 ms for HD ECoG (at the sampling rate of 2000 Hz) using a Hann window function. A default value for the window overlap equal to 25% was used. The audio spectrogram is shown in the frequency range that matches the ECoG spectrogram. The ECoG signal above 500 Hz is almost zero due to the built-in bandpass filtering of the signal in the recording system (set at 0.3–500 Hz, see Methods). Prior to calculation of the spectrogram the ECoG signal was notch-filtered to account for the line noise (at 50 Hz and all its harmonics). No other preprocessing was applied. The panel under the spectrograms shows a correlation plot for the same electrode and the audio, calculated over non-overlapping fragments of 30 seconds (given the film duration of 78 minutes there were 156 non-overlapping 30-second fragments in total). Spearman correlation (for consistency will all other analyses) was computed per each 30-second fragment of the film for each frequency bin of the electrode with that of the audio signal. To make this calculation possible, the audio spectrogram was recomputed on the audio signal at 16000 Hz at windows of 256 ms to match the temporal and frequency resolution of the ECoG spectra. Plots in the right panel show the correlation profiles across all electrodes before applying a common average reference and after. The plots show Spearman correlation values averaged over all 30-second fragments. Bottom plots show the results of the analyses from **Figures 1b** and **2a** reproduced with a HFB signal computed in the range of 60 to 90 Hz. In their original paper Roussel et al., 2019 showed that in a subject whose data were not contaminated with audio the results were well reproducible using a more limited HFB range from 60 to 90 Hz. Similar to that, we show that our results still hold when only a subset (60 – 90 Hz) of HFB signal is used.
